# The impact of GLP-1 receptor agonists on chemotherapeutic efficacy: insights from GWAS and experiments

**DOI:** 10.3389/fphar.2026.1957757

**Published:** 2026-10-23

**Authors:** Sha Huang, Yongqi Feng, Yeling Liu, Xiaochun Mo, Haichuan Hu, Haolin Chen, Mengyi Han, Wanting Huang, Wantong Zheng, Xinting Hou, Runfeng Wen, Chenbing Liu, Jiahui Wang, Yunzhen Wang

**Affiliations:** 1 Department of Thoracic Surgery, Sir Run Run Shaw Hospital, Zhejiang University School of Medicine, Hangzhou, China; 2 Nanshan School, Guangzhou Medical University, Guangzhou, China; 3 Department of Rheumatology and Clinical Immunology, Peking Union Medical College Hospital, Beijing, China; 4 The Third School of Clinical Medicine, Guangzhou Medical University, Guangzhou, China; 5 Department of Otorhinolaryngology, The Seventh Affiliated Hospital of Sun Yat-Sen University, Shenzhen, China; 6 Department of Gastroenterology, Chongqing Key Laboratory of Digestive Malignancies, Daping Hospital, Chongqing, China; 7 The Sixth School of Clinical Medicine, The Affiliated Qingyuan HospitaI (Qingyuan PeopIe’s HospitaI), Guangzhou Medical University, Guangzhou, China; 8 Department of General Surgery, Sir Run-Run Shaw Hospital, Zhejiang University, Hangzhou, China; 9 Department of Head and Neck Surgery, Sir Run Run Shaw Hospital, Zhejiang University, Hangzhou, China

**Keywords:** glucagon-like peptide-1 receptor agonist, chemotherapy, cancer, animal experiment, Mendelian randomization

## Abstract

**Background:**

Emerging evidence highlights the anti-tumor properties of Glucagon-like peptide-1 receptor agonists (GLP-1RAs). However, their synergistic potential with standard chemotherapy remains largely unexplored.

**Methods:**

Data from Genome-wide association study (GWAS) were used to select single-nucleotide polymorphisms (SNPs) strongly associated with lipid-lowering drug targets as instrumental variables. Causal effects were assessed primarily using the inverse variance weighted (IVW) method, supported by comprehensive sensitivity analyses to control for pleiotropy and heterogeneity. These Mendelian randomization (MR) findings were further validated through *in vitro* functional assays (cell viability, apoptosis, and Western blot) and an *in vivo* tumor-bearing mouse model.

**Results:**

Glucagon-like peptide-1 receptor (GLP-1R) activity showed a significantly positive causal relationship with dihydrofolate reductase (DHFR; β = 0.323, P = 6.02 × 10^−17^) and apoptosis regulator Bcl-2 (β = 0.259, P = 2.1 × 10^−11^) but a negative relationship with ribonucleoside-diphosphate reductase large subunit (RRM1; β = −0.12, P = 2.32 × 10^−3^). Functional experiments validated these genetic findings. GLP-1RA significantly enhanced the antitumoral efficacy of gemcitabine both *in vitro* and *in vivo* by repressing RRM1. Conversely, GLP-1RA attenuated the anti-tumor effects of methotrexate and paclitaxel by upregulating DHFR and Bcl-2, respectively. Notably, GLP-1RA combination therapy achieved these modulatory effects without exacerbating chemotherapy-associated systemic toxicity.

**Conclusion:**

GLP1-RAs can influence the efficacy of chemotherapeutic drugs such as methotrexate, paclitaxel, and gemcitabine.

## 1 Introduction

Glucagon-like peptide-1 receptor agonists (GLP-1RAs) are a novel class of antihyperglycemic drugs that target the Glucagon-like peptide-1 receptor (GLP-1R). GLP-1RAs have been shown to be a promising pharmacological intervention for the treatment of obesity and its related complications. In recent years, GLP-1RAs have proven effective in weight reduction [see (Wong et al., 2025)]. The U.S. Food and Drug Administration approved them for long-term weight management in 2014. The binding of GLP-1R to its ligand GLP-1 can induce changes in lipid metabolism [see (Niu et al., 2022; Bu et al., 2024)]. In addition to their established glycemic and lipid-lowering benefits, GLP-1RAs have demonstrated broader pleiotropic effects across various diseases [see (Drucker, 2026)]. Notably, an increasing body of evidence suggests that GLP-1RAs can reduce the risk of multiple malignancies [see (Dai et al., 2025; Wang et al., 2024)]. Experiments have demonstrated that GLP-1R inhibits tumor cell proliferation and promotes their apoptosis [see (Fidan-Yaylalı et al., 2016; Zhao et al., 2018)]. Semaglutide is a classic and representative agent of GLP-1RAs.

Cancer is a major public health concern with a substantial socioeconomic burden worldwide. It is one of the three leading causes of death among individuals aged 30–69 years in 177 of 183 countries [see (Bray et al., 2021)] and the second leading cause of death in the United States [see (Siegel et al., 2025)]. Chemotherapy remains a cornerstone of traditional cancer treatment. Agents such as methotrexate [see (Gradishar et al., 2024)], doxorubicin [see (Shah et al., 2024)], paclitaxel [see (Riely et al., 2024)], and gemcitabine [see (Riely et al., 2024)] exert antitumor effects against acute lymphoblastic leukemia and non-small cell lung cancer through different mechanisms of action. However, the emergence of chemoresistance further reduces the effectiveness of treatment. In recent years, numerous studies have focused on the use of hypoglycemic drugs in cancer treatment, and it has been discovered that the combination of metformin with chemotherapy can benefit patients with lung cancer[see (Nazemiyeh et al., 2025; Ndembe et al., 2024)], breast cancer [see (Mouna et al., 2025)], and endometrial cancer [see (Piffoux et al., 2025)], which prompted us to hypothesize whether the adjunctive use of GLP-1RAs with standard chemotherapy could synergistically enhance antitumor efficacy. To date, few studies have explored this combinatorial approach.

In this study, we employed Mendelian randomization (MR) to investigate whether GLP-1RA influence the efficacy of chemotherapy and whether reverse causality exists between the effects of the two types of therapies. Meanwhile, we carried out laboratory experiments to validate the findings of the MR analysis, using semaglutide as a representative GLP-1RA. The combined effects of semaglutide and standard chemotherapeutic agents were evaluated through *in vitro* analyses (cell viability, flow cytometry, and Western blot) and an *in vivo* subcutaneous tumor-bearing mouse model. The results initially indicate that semaglutide may enhance the antitumoral and apoptotic efficacy of gemcitabine by repressing ribonucleoside-diphosphate reductase large subunit (RRM1), while paradoxically attenuating the tumor-suppressive effects of methotrexate and paclitaxel by upregulating dihydrofolate reductase (DHFR) and Bcl-2. Furthermore, animal models strictly confirmed these divergent therapeutic outcomes and verified that the addition of semaglutide did not exacerbate systemic chemotherapy toxicity.

## 2 Methods

### 2.1 Study design of MR analysis

We performed bidirectional MR analysis to evaluate the causal relationship between the protein levelsof GLP-1R, and chemotherapy targets [see (Skrivankova et al., 2021)]. MR involves the use of genetic variants as instrumental variables (IVs), inherently reducing confounding bias and strengthening causal inference [see (Burgess et al., 2013)]. Our methodology rigorously adhered to the three core assumptions of MR [see (Emdin et al., 2017)]. In this study, genetic variants associated with the exposure were selected based on the genome-wide significance threshold of P < 1 × 10^−5^, and pleiotropy-robust estimates were validated through sensitivity analysis. MR uses the quasi-random nature of genetic inheritance to emulate randomized trial conditions in observational settings, enhancing the validity of causal inference.

### 2.2 Data source

Genetic association data were obtained from two public repositories that maintain rigorous curation standards: the IEU OpenGWAS Project (https://gwas.mrcieu.ac.uk/), a platform that provides harmonized summary statistics from genome-wide association study (GWAS with standardized metadata for reproducibility. These institutional-grade platforms aggregate anonymized genetic association data from large-scale population studies and consortia, employing stringent quality-control protocols and metadata standardization to ensure analytical reproducibility. All data included in this study were obtained from studies approved by institutional review boards. This study strictly adhered to international ethical standards governing secondary analysis of human genomic data. Detailed information regarding individual datasets, including population characteristics and phenotype definitions, is provided in Supplementary Table S1.

### 2.3 IV selection and bidirectional MR analysis

As the traditional genome-wide threshold yielded an insufficient number of SNPs, a genome-wide significance threshold of P < 1 × 10^−5^ was used to select single-nucleotide polymorphisms (SNPs) associated with GLP-1R as candidate IVs. Variants demonstrating weak instrument bias (F-statistic <10, calculated as F = [β/SE]^2^, β = effect size; SE = standard error [see (Chen et al., 2022; Bowden et al., 2019)]) were excluded [see (Pal et al., 2012)]. Subsequent clumping procedures were performed using stringent linkage disequilibrium (LD) criteria (pairwise r^2^ < 0.001 and LD < 10,000 kb) to ensure independence of retained IVs. After harmonizing the exposure-outcome allele effects and removing palindromic or strand-ambiguous SNPs, a set of instrumental variables was finally determined. Data harmonization strictly align the effect alleles of all selected SNPs to uniformly represent an increase in protein levels, ensuring a consistent directional proxy for upregulation. Finally, we retrieved the instrumental variables through LDlink (https://ldlink.nih.gov/?tab=home), a website established by the National Institutes of Health in the United States. Any instrumental variables associated with phenotypes that could simultaneously affect both the exposure and the outcome were excluded. The effectiveness of IVs was evaluated using the inverse variance weighted (IVW) [see (Burgess et al., 2017)], MR-Egger [see (Slob et al., 2017)], simple mode [see (Hartwig et al., 2017)], weighted mode [see (Hartwig et al., 2017)], and weighted median [see (Bowde et al., 2016)] methods. IVW served as the primary method for assessing causality [see (EPIC- InterAct Consortium et al., 2015)]. The false discovery rate (FDR) correction method was used for multiple hypothesis testing. A corrected P-value of <0.05 was considered statistically significant [see (Benjamini and Hochberg, 1995)]. Furthermore, a conservative criterion was applied: robust significant associations required the 95% confidence intervals (CIs) to distinctly exclude zero.

### 2.4 Sensitivity analysis

To ensure the methodological rigor of MR analysis, sensitivity analysis was performed using Cochran’s Q test, MR-Egger intercept test, and leave-one-out validation. Cochran’s Q test was used to evaluate heterogeneity across the effects of individual genetic variants, with a P-value exceeding 0.05 suggesting minimal heterogeneity [see (Greco et al., 2015)]. MR-PRESSO was used to detect and adjust for horizontal pleiotropy, which occurs when genetic variants influence the outcome through pathways independent of the exposure. This approach highlights genetic variants exhibiting pleiotropic effects while accounting for their influence on causal estimates, ensuring the reliability and validity of the results of primary analysis [see (Verbanck et al., 2018)]. The MR-Egger intercept test was used to assess horizontal pleiotropy without presuming all genetic variants serve as valid IVs. This method works by fitting a linear regression model between genetic variants and the outcome, and a statistically significant intercept indicates potential horizontal pleiotropy [see (Bowden et al., 2015)]. Leave-one-out analysis was used to evaluate the extent of the effect of each genetic variant on causal estimates. In this method, each variant is systematically excluded at a time and the results are recalculated, allowing the evaluation of the contribution of each genetic marker to the overall findings [see (Burgess et al., 2017)]. MR analysis was performed using the R packages “TwoSample MR” and “patchwork”. All statistical analyses and data visualization were performed using the R software (version 4.3.1). What’s more, AI was not used in the research and manuscript development according to the TITAN Guideline [see (Riaz et al., 2025)].

### 2.5 Cell culture

Human lung cancer cells A549 (Bogoo Biotechnology, Shanghai, China), MDA-MB-231 (Shanghai Zhong Qiao Xin Zhou Biotechnology Co., Ltd., Shanghai, China), and COLO205 (Wuhan Pricella Biotechnology Co., Ltd., Wuhan, China) were maintained in RPMI 1640 medium (CORNING, Mediatech, Inc., Manassas, VA, United States) supplemented with 10% fetal bovine serum (FBS, PAN-Biotech, Aidenbach, Germany) in a humidified atmosphere with 5% CO_2_. Cell lines were authenticated by Biowing Applied Biotechnology Co., Ltd. (A549) and Shanghai Zhong Qiao Xin Zhou Biotechnology Co., Ltd. (MDA-MB-231) and Wuhan Pricella Biotechnology Co., Ltd. (COLO205) via short tandem repeat profiling.

### 2.6 Reagents and antibodies

Semaglutide (HY-114118), methotrexate disodium (HY-14519A), paclitaxel (HY-B0015) and gemcitabine hydrochloride (HY-B0003) were purchased from MCE (MCE, China). The antibodies used for immunoblotting, DHFR (CY6953), Bcl-2 (CY5032) and RRM1 (CY8733) were purchased from Abways Biotechnology (Shanghai, China). Stock solutions were reconstituted by dissolving 10 mg semaglutide in 2.431 mL DMSO (1 mM), 100 mg methotrexate disodium in 40.128 mL sterile water (5 mM), 50 mg paclitaxel in 11.711 mL DMSO (5 mM), and 100 mg gemcitabine hydrochloride in 13.348 mL DMSO (25 mM). All solutions were filtered (0.22 μm) and stored at −80 °C. Working solutions were freshly prepared in complete medium prior to each assay, with the final DMSO concentration maintained at <0.1% (v/v) across all wells. Negative control (NC) cells were treated with vehicle-matched medium (0.1% DMSO/water).

### 2.7 IC50 Curves

The concentration inhibiting cell viability by 50% (IC50) was calculated by a standard curve of cell viability. Cell viability was measured with a Cell Counting Kit-8 (CCK-8) assay (Dojindo, Japan). Tumor cells were seeded in 96-well plates overnight at a density of 5,000 cells per well, and then treated with drugs under the indicated conditions and times. Next, 10% of the final volume of CCK-8 solution was added to each well and incubated at 37 °C for 1 h. A SpectraMax M4 microplate reader (Molecular Devices, LLC, Sunnyvale, CA, United States) was used to measure the absorbance at a wavelength of 450 nm. The IC50 curves were calculated using variable slope for log(inhibitor) vs. normalized response (Graphpad Prism 10.3, Boston, MA). For each cell line, the assay was independently performed in three biological replicates (n = 3), with three technical replicates (wells) per concentration in each experiment.

### 2.8 Cell apoptosis assay

The distribution of apoptotic cells was evaluated using Annexin V-FITC/PI staining (Beyotime, Shanghai, China). After indicated treatments, tumor cells were trypsinized in 0.25% trypsin with Ethylenediaminetetraacetic acid free, washed with cold phosphate-buffered saline (PBS), and then suspended in 100 μL PBS. Subsequently, the cells were stained with 5 μL of Annexin V-FITC and 10 μL of PI, before being incubated at room temperature for 30 min in the dark. The stained cells were visualized by flow cytometry (FACS CantoII, BD Biosciences, United States) and analyzed using Flowjo (FlowJo 10.4, LLC, United States). All flow cytometry assays were independently repeated in three biological replicates (n = 3). Quantitative data are presented as mean ± standard deviation (SD) across the three independent experiments.

### 2.9 Western blot

Cells were first washed with ice-cold PBS and then lysed in RIPA lysis buffer (Beyotime, Shanghai, China). Sonication was used to reduce the viscosity of the lysate, and a BCA assay (Solarbio, China) was carried out to measure the protein concentration. The lysates were then mixed with 3X reducing SDS loading buffer (#7723, Cell Signaling Technology, Inc., United States) and denatured at 100 °C for 5 min. The lysates were separated with 10%–12% sodium dodecyl sulfate polyacrylamide gel electrophoresis (SDS-PAGE) gels and then transferred onto a polyvinylidene difluoride (PVDF) membrane (Millipore, Billerica, MA). The membrane was blocked with 5% nonfat milk for 1 h at room temperature and incubated overnight at 4 °C with primary antibodies. After that, the membrane was washed 3 times with TBST (tris-buffered saline, 0.1% Tween-20) and then probed with a secondary antibody for 1 h. After washing with TBST, the blots were incubated with a high-sensitivity chemiluminescence detection system (Millipore) and visualized using the Amersham Biosciences ECL Detection System (Amersham plc, GE Healthcare, Chicago, IL, United States). Western blot experiments were conducted with at least three independent biological replicates (n = 3). Representative images from one replicate are shown. For densitometric quantification, protein bands from three independent biological replicates (n = 3) were analyzed using Image Lab software (Version 6.0; Bio-Rad Laboratories, Inc.). The optical density of target protein bands (RRM1, DHFR, and Bcl-2) was normalized against that of the corresponding GAPDH loading control.

### 2.10 Xenograft model and treatments

All animal experiments were approved by the Institutional Animal Care and Use Committee of Zhejiang Center of Laboratory Animals (Approval No. ZJCLA-IACUC-20011471). Each animal represented one experimental unit. The sample size was calculated using the animal sample size calculator (http://www.lasec.cuhk.edu.hk/sample-size-calculation.html). Based on preliminary data for xenograft tumor volume variation, with a two-sided significance level (α) of 0.05, a statistical power (1−β) of 80%, and an anticipated effect size (δ/σ ≥ 2.0) for distinguishing combination synergism/antagonism from monotherapy, a minimum sample size of five animals per group was required. The total number of animals used in the *in vivo* experiments was 80, which were divided into two parallel cohorts (40 mice per cohort; eight groups of n = 5 each) to separate tumor burden analysis from survival evaluation. In Cohort 1, animals received the indicated drug regimens for 28 days. Tumor volumes were longitudinally measured via calipers. At day 28, all mice in this cohort were humanely euthanized by cervical dislocation according to the American Veterinary Medical Association Guidelines (Schaumburg, IL, United States; Supplementary Figure S1); tumors were carefully excised, photographed, and weighed. In Cohort 2, an identical set of animals under the identical experimental conditions and treatments was maintained exclusively for long-term Kaplan-Meier survival analysis. Animals were monitored daily and euthanized upon reaching predetermined humane endpoints (e.g., tumor diameter exceeding 15 mm, ulceration, or weight loss >20%), which was recorded as the survival endpoint. There were no specific inclusion and exclusion criteria for animals. All animals were included for the final analysis. Five-week-old male BALB/c nude mice (Shanghai Sippr-bk Laboratory Animal Co., Ltd., China; weight, 16–20 g) were injected subcutaneously in the right flanks with 3 × 10^6^ LLC cells mixed 1:1 with 100% Matrigel. On day 8, when tumors had reached 40–80 mm^3^, the mice were separated into eight groups (5 mice per group), and animals were randomly allocated to different groups using a Research Randomizer (https://www.randomizer.org/). All studies were performed with the blinding of the experiment operator. Experimental and control animals were kept in mixed cages to reduce confounding variables. All animal experiments were performed at least twice.

Animals were grouped as follows (Wong et al., 2025). Con, subcutaneous injection of water (50 μL/10 g, three times per week) + intraperitoneal saline (100 μL/10 g, twice a week) (Niu et al., 2022); semaglutide, subcutaneous injection of semaglutide (100 μg/kg, three times per week) + intraperitoneal saline (100 μL/10 g, twice a week) (Bu et al., 2024); Methotrexate (MTX), subcutaneous injection of water (50 μL/10 g, three times per week) + intraperitoneal methotrexate (5 mg/kg, twice a week) (Drucker, 2026); MTX + semaglutide, subcutaneous injection of semaglutide (100 μg/kg, three times per week) + intraperitoneal methotrexate (5 mg/kg, twice a week) (Dai et al., 2025); Paclitaxel (PTX), subcutaneous injection of water (50 μL/10 g, three times per week) + intraperitoneal paclitaxel (10 mg/kg, twice a week) (Wang et al., 2024); PTX + semaglutide, subcutaneous injection of semaglutide (100 μg/kg, three times per week) + intraperitoneal paclitaxel (10 mg/kg, twice a week) (Fidan-Yaylalı et al., 2016); Gemcitabine (Gem), subcutaneous injection of water (50 μL/10 g, three times per week) + intraperitoneal gemcitabine (25 mg/kg, twice a week) (Zhao et al., 2018); Gem + semaglutide, subcutaneous injection of semaglutide (100 μg/kg, three times per week) + intraperitoneal gemcitabine (25 mg/kg, twice a week). The tumor volume was calculated using the following formula: volume = 1/2(length × width^2^). Statistical analyses were performed using Prism (Graphpad Prism 10.3, Boston, MA). Longitudinal tumor volume progression and mouse body weight changes over time were analyzed using two-way repeated-measures ANOVA (or a mixed-effects model if missing values occurred) followed by Tukey’s post-hoc test for multiple comparisons, with treatment group as the between-subject factor and time as the within-subject factor. Terminal excised tumor weights at Day 28 were compared using standard one-way ANOVA with Tukey’s post-hoc test. Overall survival was assessed using Kaplan–Meier survival curves and compared via the Log-rank (Mantel–Cox) test.

## 3 Result

### 3.1 IV selection

We identified 31 SNPs associated with GLP-1R through stringent methodological screening. Correspondingly, for the reverse MR analysis, instrumental variables were also selected for the four chemotherapy targets serving as exposures. Detailed information is shown in Supplementary Table S2. All SNPs demonstrated strong association signals (F-statistic >10), effectively mitigating weak instrument bias. In addition, these SNPs met the genome-wide significance threshold (P < 1 × 10^−5^) and LD clumping (r^2^ < 0.001) criteria, which ensured their independence and biological relevance. The detailed characteristics of the selected SNPs, including chromosomal locations, effect alleles, and association statistics, are summarized in Supplementary Table S2.

### 3.2 MR revealed that GLP-1R has a positive causal association with DHFR and Bcl-2, and a negative association with RRM1

The results of bidirectional MR are summarized in Figure 1. MR analysis revealed significant associations between genetically predicted GLP-1R and the protein levels of chemotherapy targets. IVW demonstrated a positive causal relationship between GLP-1R and DHFR protein levels (β = 0.323 [0.248–0.399]; P = 6.02 × 10^−17^). This positive relationship was corroborated by the results of weighted median (β = 0.291 [0.180–0.402]; P = 2.77 × 10^−7^) and MR-Egger analysis (β = 0.340 [0.157–0.522]; P = 1.02 × 10^−3^). Furthermore, GLP-1R showed a positive relationship with the protein levels of the apoptosis regulator Bcl-2 (β = 0.259 [0.183–0.335]; P = 2.1 × 10^−11^) but a negative relationship with the protein levels of RRM1 (β = −0.12 [−0.197 to −0.043]; P = 2.32 × 10^−3^). IVW revealed no significant association between GLP-1R and DNA topoisomerase 1 (β = −0.079 [−0.158 to 0]; P = 0.049) in Figure 2.

**FIGURE 1:**
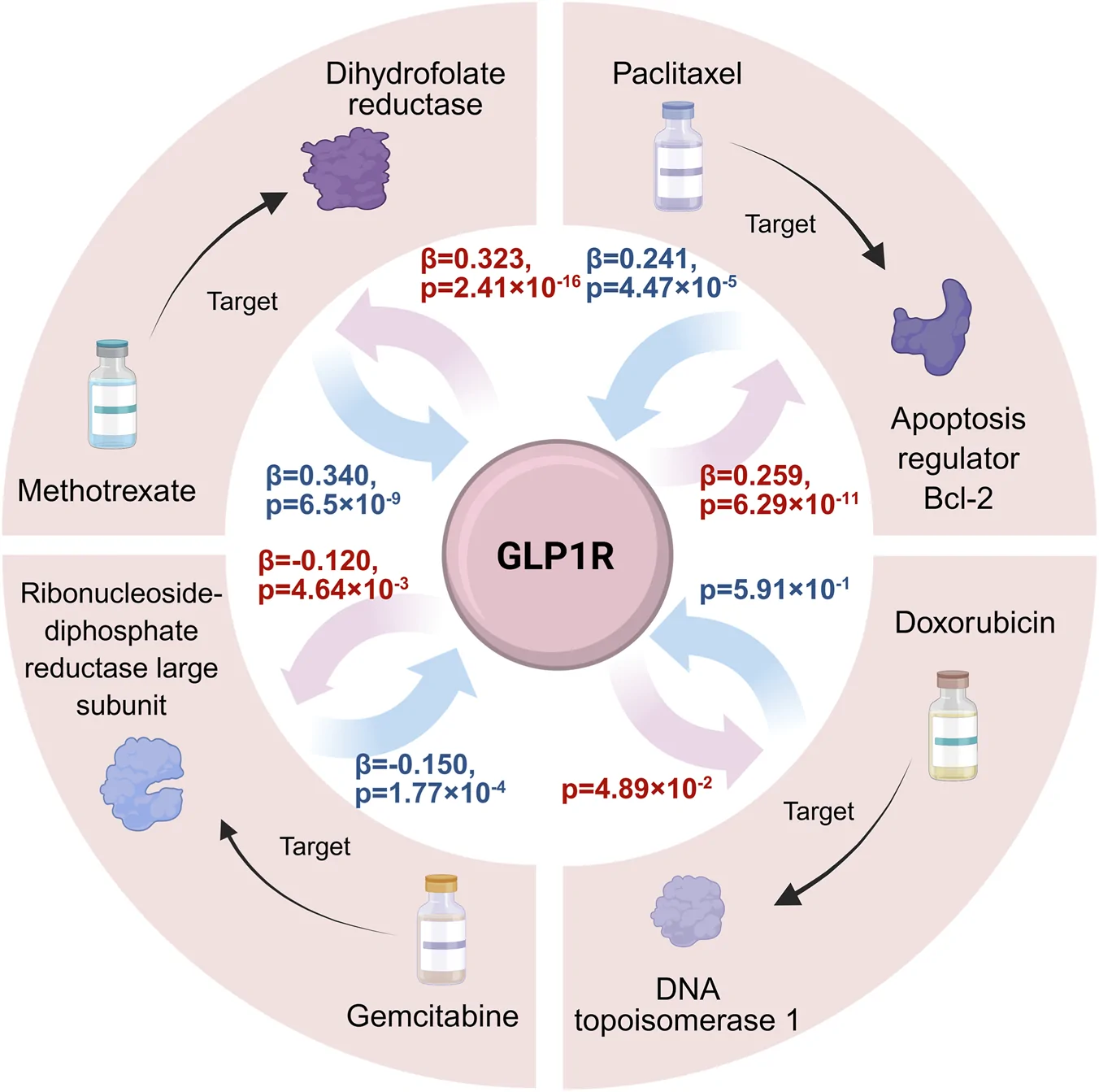
Summary of results of bidirectional Mendelian randomization. Red arrow, forward Mendelian randomization; Blue arrow, reverse Mendelian randomization; β, beta; p, p value.

**FIGURE 2:**
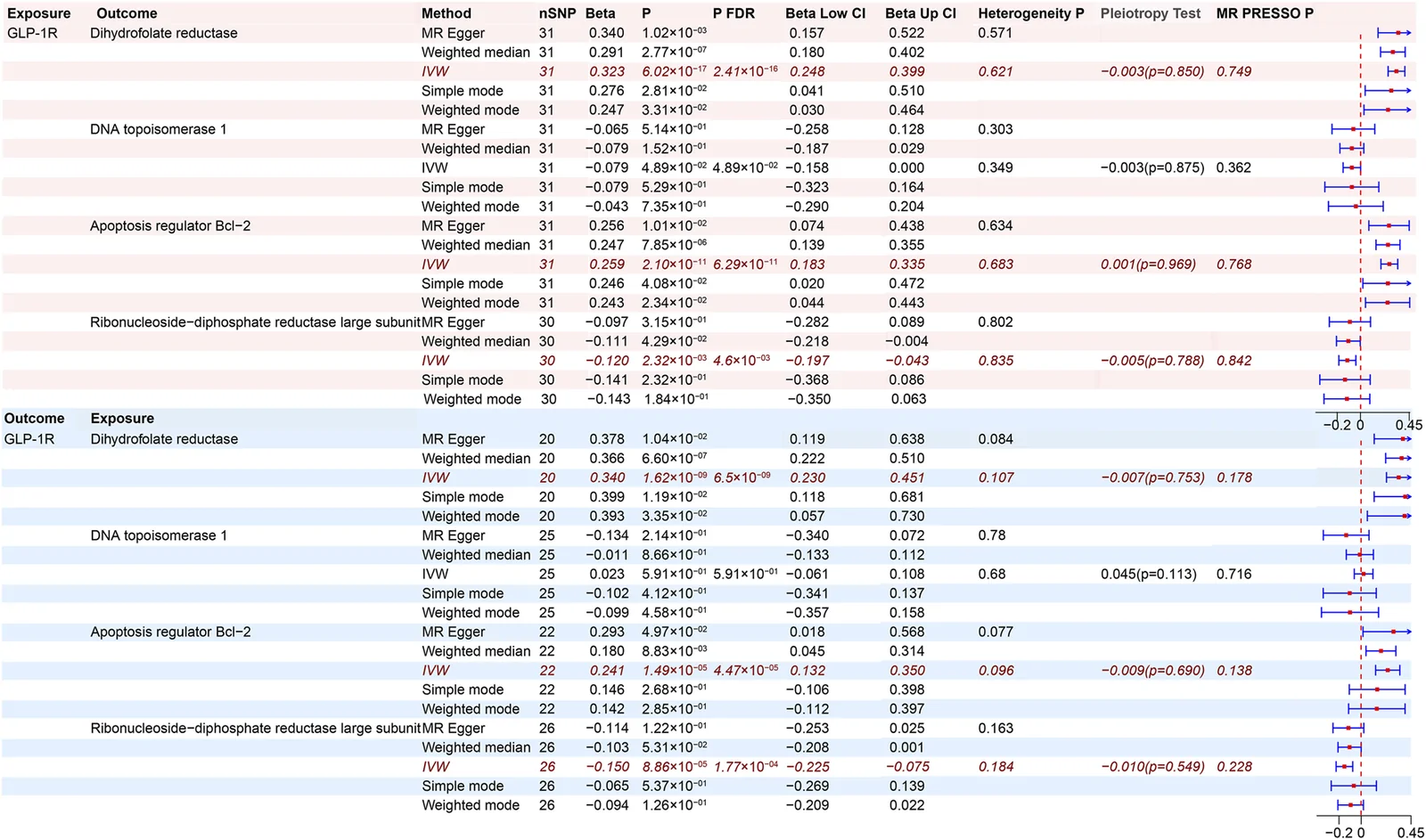
Association of gene replacement GLP-1R with protein levels targeted by chemotherapeutic agents. CI, confidence interval.

The MR-Egger intercept test (P *>* 0.05) and MR-PRESSO analysis showed no evidence of horizontal pleiotropy in Supplementary Figure S2, whereas Cochran’s Q test (P *>* 0.05) indicated minimal heterogeneity in Figure 2. Leave-one-out analysis demonstrated the stability of the results in Supplementary Figure S3, with forest and funnel plots showing no influential outliers in Supplementary Figures S4, 5.

### 3.3 Reverse MR indicated a positive association between DHFR levels and genetically predicted GLP-1R

In reverse MR analysis, MR-PRESSO revealed one pleiotropic outlier, which was excluded before the final modeling. Primary analysis through IVW showed that the protein levels of DHFR were positively associated with genetically predicted GLP-1R (β = 0.340 [0.230–0.451]; P = 1.62 × 10^−9^). Furthermore, apoptosis regulator Bcl-2 levels were positively associated with GLP-1R (β = 0.241 [0.132–0.350]; P = 1.49 × 10^−5^), whereas RRM1 protein levels were negatively associated with GLP-1R (β = −0.150 [−0.225 to −0.075]; P = 8.86 × 10^−5^). No significant associations between other chemotherapy targets and GLP-1R were detected via IVW (P = 0.591) in Figure 2.

MR-Egger regression revealed no evidence of horizontal pleiotropy (P > 0.05) in Supplementary Figures S6, and Cochran’s Q test confirmed the absence of heterogeneity (P > 0.05) across IVs in Figure 2. Leave-one-out analysis validated the stability of causal estimates in Supplementary Figures S7, with forest and funnel plots demonstrating symmetrical effect distributions in Supplementary Figures S8, 9.

### 3.4 Semaglutide can enhance the antitumoral treatment efficacy of gemcitabine, while weakening the anti-tumor effects of methotrexate and paclitaxel

The effects of semaglutide combination treatments on the efficacy of chemotherapeutic drugs were analyzed in three different tumor cells: A549 (human lung cancer cell lines), MDA-MB-231 (human breast cancer cell lines), COLO205 (human colorectal adenocarcinoma cell lines). As shown in Figure 3A, compared to single gemcitabine treatment, combination treatment with semaglutide clearly decreased the IC50 values of A549 (from 94.8 to 23.3 nM), MDA-MB-231 (2302–325.1 nM) and COLO205 (588.3–180.5 nM. However, semaglutide increased the IC50 values of methotrexate and paclitaxel in A549 (from 52.4 to 203 nM, MTX; from 0.01 to 0.1 nM, PTX), MDA-MB-231 (from 6637 to 8375 nM, MTX; from 0.01 to 0.07 nM, PTX) and COLO205 (from 368.9 to 485.3 nM, MTX; from 0.03 to 0.26, PTX).

**FIGURE 3:**
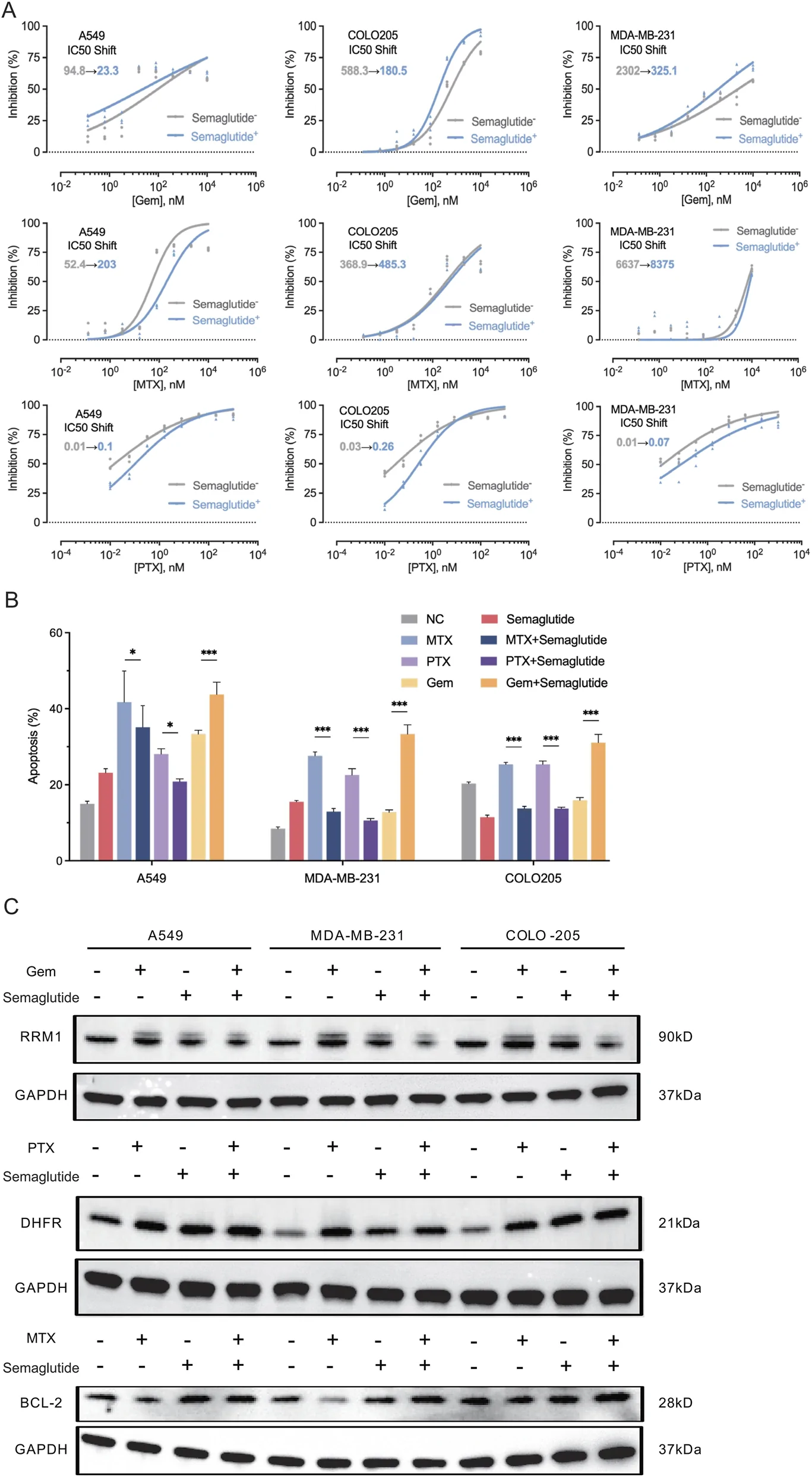
Semaglutide exhibited different effects on chemotherapeutic agents in the *in vitro* antitumoral experiments. **(A)** Half maximal inhibitory concentration (IC50) curves for single chemotherapeutic agents and semaglutide combination therapy in three different tumor cell lines were determined using the CCK-8 assay. A549, MDA-MB-231 and COLO205 cells were treated with semaglutide (1 μM) and/or gemcitabine (Ranging from 0.13 to 10,000 nM in a 5-fold serial dilution series), methotrexate (Ranging from 0.13 to 10,000 nM in a 5-fold serial dilution series) and paclitaxel (Ranging from 0.01 to 1000 nM in a 5-fold serial dilution series) separately for 72 h. **(B)** A549, MDA-MB-231 and COLO205 cells were treated with semaglutide (1 μM) and/or gemcitabine (10 μM), methotrexate (10 μM) and paclitaxel (10 nM) separately for 24 h. Then, the cells were examined using Annexin-FITC/PI staining, and the distribution of apoptotic cells was measured by flow cytometry analysis. **(C)** Representative Western blot images showing the protein levels of RRM1, DHFR and Bcl-2 in A549, MDA-MB-231 and COLO205 cells after treatment with semaglutide (1 μM) and/or gemcitabine (10 μM), methotrexate (10 μM) and paclitaxel (10 nM) for 24 h. GAPDH was used as an internal control. Data are representative of three independent biological replicates (n = 3). Error bars represent mean ± SD (n = 3). *, P < 0.05, ***, P < 0.001, by two-way analysis of variance followed by Tukey’s multiple comparisons test. NC, negative control; MTX, methotrexate; PTX, paclitaxel; Gem, gemcitabine.

Next, the effect of semaglutide on chemotherapy-induced apoptotic tumor cells was evaluated by Flow cytometry in Supplementary Figures S10, which showed that combination treatment with GLP-1R significantly increased the number of apoptotic cells in three different tumor cell lines (the total percentage of cells in quadrants Q2 + Q3) compared to that following treatment with gemcitabine alone, while the methotrexate or paclitaxel induced apoptosis of tumor cells was significantly decreased by combination treatment with semaglutide in Figure 3B.

To investigate the role of semaglutide in the corresponding targets of gemcitabine, methotrexate and paclitaxel, we used Western blot to detect the expression levels of RRM1 (A target of gemcitabine), DHFR (A target of methotrexate) and Bcl-2 (A target of paclitaxel). To quantitatively substantiate the molecular target alterations, densitometric analysis normalized to GAPDH was performed across three independent biological replicates. As shown in Figure 3C and Supplementary Figures S11, the relative expression of RRM1 in all three tumor cell lines were indeed significantly downregulated following semaglutide treatment compared to the vehicle control. Conversely, semaglutide markedly elevated Bcl-2 expression, and co-treatment with semaglutide and methotrexate resulted in a significant upregulation of DHFR across all three cell lines.

### 3.5 Semaglutide exhibited different effects on chemotherapeutic agents in the *in vivo* tumor suppression experiments of lung cancer

To evaluate the therapeutic potential of semaglutide in combination with standard-of-care chemotherapeutic agents, we employed a subcutaneous LLC-bearing model using male BALB/c nude mice divided into two parallel cohorts (Cohort one for tumor burden evaluation and Cohort 2 for long-term survival monitoring; n = 5 per group per cohort). Figure 4A shows the overall experimental timeline for *in vivo* therapeutic effect evaluation. In Cohort 1, the tumor suppressive effects varied significantly depending on the specific chemotherapy partner used. Tumor volume expanded more rapidly in the MTX + semaglutide group than in the MTX monotherapy group (p < 0.001); likewise, the addition of semaglutide to PTX resulted in a marked loss of tumor control compared to PTX alone (Figure 4B). While gemcitabine alone significantly slowed tumor progression compared to the NC group, the addition of semaglutide resulted in near-complete stagnation of tumor growth (p < 0.05 vs. Gem alone; Figure 4B). Consistent with the longitudinal tumor growth curves, macroscopic evaluation of tumors harvested at Day 28 clearly demonstrates that combination treatment with semaglutide synergistically reduced tumor size when paired with gemcitabine (Gem + semaglutide) (Figure 4C). Semaglutide monotherapy showed no significant reduction in tumor weight compared to the negative control group, suggesting limited independent anti-tumor activity at the administered dose. Conversely, tumor weights at Day 28 were significantly higher in the combination groups than in the respective monotherapy groups when semaglutide combined with methotrexate or paclitaxel (p < 0.001 for MTX; p < 0.05 for PTX; Figure 4D). A potent synergistic effect was confirmed with gemcitabine and semaglutid, resulting in the most profound reduction in tumor volume and weight among all experimental groups (p < 0.05 vs. Gem alone; Figures 4B,D).

**FIGURE 4:**
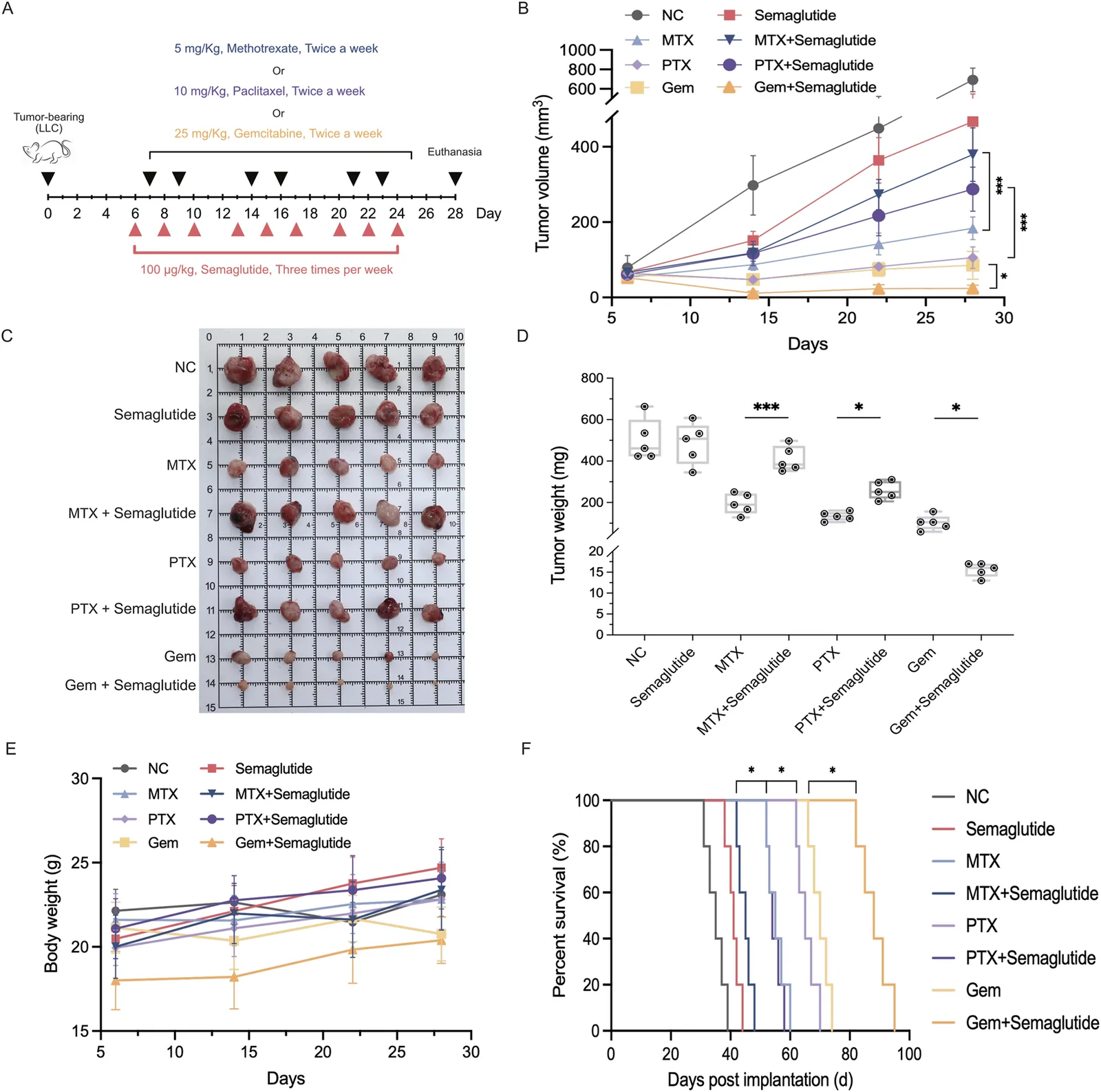
Enhancing and attenuating Effects of semaglutide with chemotherapeutics agents *in vivo*. **(A)** Schematic representation of the *in vivo* experiment. **(B)** Tumor sizes in the eight groups were recorded with a caliper measurement and calculated. **(C)** Macroscopic appearance of excised tumors from the subcutaneous LLC mouse model across different treatment groups. **(D)** Tumor weights of subcutaneous tumors excised from Cohort one at Day 28 (n = 5 per group). **(E)** The body weight change curve of mice after administration. **(F)** Kaplan-Meier survival curves of LLC tumor-bearing mice in an independent survival cohort (Cohort 2, n = 5 per group) followed up to the humane endpoint. Points, error bars and connecting lines on the graph indicate the means and error ± SDs. The box graph indicates the minimum, first quartile, median, third quartile, and maximum. The bar graph indicates the means ± SDs. *, P < 0.05, **, P < 0.001. Tumor growth curves and body weight changes were analyzed by two-way repeated-measures ANOVA with Tukey’s post-hoc test (time × treatment interaction); Terminal tumor weights were analyzed by one-way ANOVA followed by Tukey’s post-hoc test. NC, negative control; MTX, methotrexate; PTX, paclitaxel; Gem, gemcitabine.

Across the 28-day treatment course in Cohort 1, no significant body weight loss (>10−20%) was observed in any treatment group, suggesting that the addition of semaglutide did not exacerbate the systemic toxicity commonly associated with cytotoxic chemotherapeutic agents (Figure 4E). Furthermore, in the dependent survival tracking cohort (Cohort 2), the median survival duration of mice treated with Gem + semaglutide was 88 days, which was significantly prolonged compared to mice treated with Gem alone (70 days; P < 0.05; Figure 4F). Conversely, combining semaglutide with PTX significantly reduced the median survival compared to PTX monotherapy (54 vs. 65 days; P < 0.05). Similarly, the MTX + semaglutide group exhibited accelerated progression and earlier mortality relatice to the MTX-only group (45 vs. 55 days; P < 0.05; Figure 4F).

## 4 Discussion

To the best of our knowledge, this study is the first to use bidirectional MR analysis to evaluate the causal relationship between GLP-1R and chemotherapy targets at the genetic level. MR analysis showed that GLP-1R was positively associated with the protein levels of DHFR and apoptosis regulator Bcl-2 but negatively associated with the protein levels of RRM. Reverse MR analysis showed that the protein levels of DHFR and apoptosis regulator Bcl-2 were positively associated with GLP-1R, whereas those of RRM1 were negatively associated with GLP-1R.

Methotrexate, an important chemotherapeutic drug for leukemia, targets DHFR to exert antitumor effects [see (Pollyea et al., 2023)]; paclitaxel targets apoptosis regulator Bcl-2 to activate apoptosis[see (Ferlini et al., 2009)]; and gemcitabine, a deoxycytidine analogue, covalently binds to and inactivates the large subunit of ribonucleotide triphosphate reductase to inhibit DNA replication[see (Wang et al., 2007)]. By browsing DRUGBANK (https://go.drugbank.com), we found that doxorubicin, which targets DNA topoisomerase 1, can also affect DNA replication. On the basis of this study, we recommend that GLP-1RAs, such as semaglutide, should not be used concurrently with methotrexate or paclitaxel for cancer treatment, as GLP-1RAs may promote tumor cell proliferation and increase the tumor burden. On the contrary, GLP-1RAs can be used as adjunctive drugs when using gemcitabine. Reverse MR analysis showed significant causal relationships between the three chemotherapy targets and GLP-1R. These findings suggest that an increase in GLP-1R levels increases DHFR levels, and an increase in DHFR levels promotes an increase in GLP-1R levels, forming a positive feedback loop. However, the population of this study focused on Europe, and the populations of other countries and regions were not described. Therefore, the universality of the results of this study is insufficient. We look forward to obtaining data from other regions in the future and conducting further research. Although we accounted for several potential confounding factors in the MR analysis, residual confounding due to unmeasured or unknown genetic and environmental factors cannot be entirely ruled out, potentially introducing bias into the causal estimates. While MR served as an effective initial screening strategy to identify potential chemotherapeutic targets downstream of GLP-1R, we acknowledge that genetic variants cannot fully recapitulate pharmacological effects. Therefore, our *in vitro* and *in vivo* experiments were indispensable to empirically validate target modulation.

We performed *in vitro* viability and apoptosis assays. These experiments demonstrated that semaglutide combination therapy profoundly enhanced the cytotoxicity of gemcitabine but paradoxically attenuated the tumor-killing effects of methotrexate and paclitaxel. The targeted protein expression analysis revealed that semaglutide selectively inhibited RRM1, while simultaneously upregulating DHFR and Bcl-2. Finally, to validate these therapeutic potentials and assess systemic safety in a complex physiological environment, we utilized an *in vivo* subcutaneous tumor model. The murine data corroborated our *in vitro* and genetic findings, confirming that semaglutide specifically potentiates gemcitabine efficacy while compromising methotrexate and paclitaxel treatments, all without exacerbating systemic toxicity. A study identified key phenotypic changes, which may support our findings: gemcitabine-resistant pancreatic cancer cells showed markedly reduced expression of GLP-1R, cAMP, and PKA. Notably, treatment with the GLP-1R agonist liraglutide reversed this downregulation and dose-dependently inhibited the proliferation and colony formation of these resistant cells [see (Zhao et al., 2020)]. Many existing studies may provide ideas for the mechanism research on the effect of semaglutide on chemotherapy. GLP-1RAs can inhibit the growth of breast cancer cells *in vitro* and *in vivo* by suppressing the activation of NF-κB but have no significant effects on the apoptosis of these cells [see (Iwaya et al., 2017; Eftekhari et al., 2020)]. The combined use of docetaxel (a chemotherapeutic drug) and liraglutide (a GLP-1RA) has been shown to reduce the viability of LNCaP cells and cause G2/M-phase arrest [see (Eftekhari et al., 2020)]. GLP-1R has been shown to inhibit the proliferative, invasive, and migratory abilities of mouse endometrial cancer cells by activating the cAMP/PKA signaling pathway [see (Li et al., 2022)]. In this study, GLP-1R was found to have a positive causal relationship with the protein levels of DHFR and a negative causal relationship with the protein levels of RRM1. Both of these chemotherapy targets are involved in DNA replication. Gemcitabine operates primarily as a mechanism-based suicide inhibitor that irreversibly traps the RRM1 catalytic site without necessarily inducing rapid protein degradation. Conversely, semaglutide actively suppressed RRM1 expression, consistent with our forward and reverse MR findings. Specifically, the combination treatment significantly triggers intracellular cAMP/PKA cascade or represses NF-κB and AKT/mTOR signaling pathways. Given that NF-κB positively regulates ribonucleotide reductase transcription, semaglutide-mediated transcriptional attenuation of the NF-κB pathway downregulates RRM1 synthesis at its source. By downregulating RRM1 protein abundance, semaglutide deprives the cancer cells of their enzymatic reserve and dNTP replenishment, thereby sensitizing tumor cells to gemcitabine-induced DNA synthesis arrest and synthetic lethality. Moving forward, we hope that more in-depth mechanistic studies will be conducted to fully elucidate how GLP-1RAs modulate the efficacy of chemotherapeutic agents. Furthermore, we highly anticipate that future randomized controlled trials (RCTs) will translate these preclinical findings into clinical practice, ultimately bringing novel therapeutic benefits to cancer patients undergoing chemotherapy.

## 5 Conclusion

This study investigated the genetic associations between GLP-1R and chemotherapeutic targets, revealing that genetically proxied GLP-1R signaling is associated with altered levels of DHFR, Bcl-2, and RRM1. Supported by *in vitro* and *in vivo* models, we observed that semaglutide combination treatment enhances the antitumoral efficacy of gemcitabine while compromising the therapeutic effects of methotrexate and paclitaxel. Importantly, while these phenotypic outcomes coincide with changes in target protein expression, the direct molecular mechanisms mediating these effects remain to be elucidated. These findings highlight a critical need for future mechanistic research into GLP-1RA-chemotherapy interactions, providing a foundational perspective for optimizing combination therapies and managing potential drug-drug interactions in oncology.

## Data Availability

The datasets presented in this study can be found in online repositories. The names of the repository/repositories and accession number(s) can be found below: https://gwas.mrcieu.ac.uk/.

## References

[B1] Benjamini Y. Hochberg Y. (1995). Controlling the false discovery rate: a practical and powerful approach to multiple testing. J. R. Stat. Soc. Ser. B Stat. Methodol. 57 (1), 289–300. 10.1111/j.2517-6161.1995.tb02031.x

[B2] Bowden J. Davey Smith G. Haycock P. C. Burgess S. (2016). Consistent estimation in Mendelian randomization with some invalid instruments using a weighted median estimator. Genet. Epidemiol. 40 (4), 304–314. 10.1002/gepi.21965 27061298 PMC4849733

[B3] Bowden J. Davey Smith G. Burgess S. (2015). Mendelian randomization with invalid instruments: effect estimation and bias detection through egger regression. Int. J. Epidemiol. 44 (2), 512–525. 10.1093/ije/dyv080 26050253 PMC4469799

[B4] Bowden J. Del Greco M. F. Minelli C. Zhao Q. Lawlor D. A. Sheehan N. A. (2019). Improving the accuracy of two-sample summary-data Mendelian randomization: moving beyond the NOME assumption. Int. J. Epidemiol. 48 (3), 728–742. 10.1093/ije/dyy258 30561657 PMC6659376

[B5] Bray F. Laversanne M. Weiderpass E. Soerjomataram I. (2021). The ever‐increasing importance of cancer as a leading cause of premature death worldwide. Cancer 127 (16), 3029–3030. 10.1002/cncr.33587 34086348

[B6] Bu T. Sun Z. Pan Y. Deng X. Yuan G. (2024). Glucagon-like Peptide-1: new regulator in lipid metabolism. Diabetes Metab. J. 48 (3), 354–372. 10.4093/dmj.2023.0277 38650100 PMC11140404

[B7] Burgess S. Butterworth A. Thompson S. G. (2013). Mendelian randomization analysis with multiple genetic variants using summarized data. Genet. Epidemiol. 37 (7), 658–665. 10.1002/gepi.21758 24114802 PMC4377079

[B8] Burgess S. Bowden J. Fall T. Ingelsson E. Thompson S. G. (2017). Sensitivity analyses for robust causal inference from mendelian randomization analyses with multiple genetic variants. Epidemiology 28 (1), 30–42. 10.1097/EDE.0000000000000559 27749700 PMC5133381

[B9] Chen L. Yang H. Li H. He C. Yang L. Lv G. (2022). Insights into modifiable risk factors of cholelithiasis: a Mendelian randomization study. Hepatology 75 (4), 785–796. 10.1002/hep.32183 34624136 PMC9300195

[B10] Dai H. Li Y. Lee Y. A. Lu Y. George T. J. Donahoo W. T. (2025). GLP-1 receptor agonists and cancer risk in adults with obesity. JAMA Oncol. 11 (10), 1186–1193. 10.1001/jamaoncol.2025.2681 40839273 PMC12371547

[B11] Drucker D. J. (2026). The expanding landscape of GLP-1 medicines. Nat. Med. 32 (1), 47–57. 10.1038/s41591-025-04124-5 41482564

[B12] Eftekhari S. Montazeri H. Tarighi P. (2020). Synergistic anti-tumor effects of liraglutide, a glucagon-like peptide-1 receptor agonist, along with docetaxel on LNCaP prostate cancer cell line. Eur. J. Pharmacol. 878, 173102. 10.1016/j.ejphar.2020.173102 32283060

[B13] Emdin C. A. Khera A. V. Kathiresan S. (2017). Mendelian Randomization. 10.1001/jama.2017.17219 29164242

[B14] EPIC- InterAct Consortium Burgess S. Scott R. A. Timpson N. J. Davey Smith G. Thompson S. G. (2015). Using published data in Mendelian randomization: a blueprint for efficient identification of causal risk factors. Eur. J. Epidemiol. 30 (7), 543–552. 10.1007/s10654-015-0011-z 25773750 PMC4516908

[B15] Ferlini C. Cicchillitti L. Raspaglio G. Bartollino S. Cimitan S. Bertucci C. (2009). Paclitaxel directly binds to Bcl-2 and functionally mimics activity of Nur77. Cancer Res. 69 (17), 6906–6914. 10.1158/0008-5472.CAN-09-0540 19671798

[B16] Fidan-Yaylalı G. Dodurga Y. Seçme M. Elmas L. (2016). Antidiabetic exendin-4 activates apoptotic pathway and inhibits growth of breast cancer cells. Tumor Biol. 37 (2), 2647–2653. 10.1007/s13277-015-4104-9 26399993

[B17] Gradishar W. J. Moran M. S. Abraham J. Abramson V. Aft R. Agnese D. (2024). Breast cancer, version 3.2024, NCCN clinical practice guidelines in oncology. J. Natl. Compr. Cancer Netw. 22 (5), 331–357. 10.6004/jnccn.2024.0035 39019058

[B18] Greco M. F. D. Minelli C. Sheehan N. A. Thompson J. R. (2015). Detecting pleiotropy in Mendelian randomisation studies with summary data and a continuous outcome. Statistics Med. 34 (21), 2926–2940. 10.1002/sim.6522 25950993

[B19] Hartwig F. P. Davey Smith G. Bowden J. (2017). Robust inference in summary data Mendelian randomization via the zero modal pleiotropy assumption. Int. J. Epidemiol. 46 (6), 1985–1998. 10.1093/ije/dyx102 29040600 PMC5837715

[B20] Iwaya C. Nomiyama T. Komatsu S. Kawanami T. Tsutsumi Y. Hamaguchi Y. (2017). Exendin-4, a glucagonlike Peptide-1 receptor agonist, attenuates breast cancer growth by inhibiting NF-κB activation. Endocrinology 158 (12), 4218–4232. 10.1210/en.2017-00461 29045658

[B21] Li W. Gu Y. Liu S. Ruan F. Lv W. (2022). GLP1R inhibits the progression of endometrial carcinoma through activation of cAMP/PKA pathway. Clin. Lab. Anal. 36 (10), e24604. 10.1002/jcla.24604 35989517 PMC9551121

[B22] Mouna B. M. Batra A. Sharma V. Kataria B. Gogia A. Sharma J. (2025). Metformin with neoadjuvant chemotherapy in localized triple‐negative and Her2neu‐positive breast cancer: a prospective phase 2 open‐label randomized controlled trial (McBETH). Cancer 131 (24), e70205. 10.1002/cncr.70205 41348549

[B23] Nazemiyeh A. Dadashi H. Mashinchian M. Karimian-Shaddel A. Mohabbat A. Eskandani M. (2025). Exploring the synergistic effects of metformin and doxorubicin loaded chitosan nanoparticles for A549 lung cancer therapy. Sci. Rep. 15 (1), 22657. 10.1038/s41598-025-07996-2 40594871 PMC12215345

[B24] Ndembe G. Intini I. Moro M. Grasselli C. Panfili A. Panini N. (2024). Caloric restriction and metformin selectively improved LKB1-mutated NSCLC tumor response to chemo- and chemo-immunotherapy. J. Exp. Clin. Cancer Res. 43 (1), 6. 10.1186/s13046-023-02933-5 38163906 PMC10759660

[B25] Niu S. Chen S. Chen X. Ren Q. Yue L. Pan X. (2022). Semaglutide ameliorates metabolism and hepatic outcomes in an NAFLD mouse model. Front. Endocrinol. 13, 1046130. 10.3389/fendo.2022.1046130 36568109 PMC9780435

[B26] Palmer T. M. Lawlor D. A. Harbord R. M. Sheehan N. A. Tobias J. H. Timpson N. J. (2012). Using multiple genetic variants as instrumental variables for modifiable risk factors. Stat. Methods Med. Res. 21 (3), 223–242. 10.1177/0962280210394459 21216802 PMC3917707

[B27] Piffoux M. Leary A. Follana P. Abdeddaim C. Joly F. Bin S. (2025). Olaparib combined to metronomic cyclophosphamide and metformin in women with recurrent advanced/metastatic endometrial cancer: the ENDOLA phase I/II trial. Nat. Commun. 16 (1), 1821. 10.1038/s41467-025-56914-7 39979249 PMC11842746

[B28] Pollyea D. A. Altman J. K. Assi R. Bixby D. Fathi A. T. Foran J. M. (2023). Acute myeloid leukemia, version 3.2023, NCCN clinical practice guidelines in oncology. J. Natl. Compr. Canc Netw. 21 (5), 503–513. 10.6004/jnccn.2023.0025 37156478

[B29] Riaz A. A. Ginimol M. Rasha R. Ahmed K. Ahmed A. Catrin S. (2025). Transparency in the reporting of artificial intelligence – The TITAN guideline. Premier Science. 1 (2), 100082. 10.70389/pjis.100082

[B30] Riely G. J. Wood D. E. Ettinger D. S. Aisner D. L. Akerley W. Bauman J. R. (2024). Non–small cell lung cancer, version 4.2024. J. Natl. Compr. Cancer Netw. 22 (4), 249–274. 10.6004/jnccn.2204.0023 38754467

[B31] Shah B. Mattison R. J. Abboud R. Abdelmessieh P. Aldoss I. Burke P. W. (2024). Acute lymphoblastic leukemia, version 2.2024, NCCN clinical practice guidelines in oncology. J. Natl. Compr. Cancer Netw. 22 (8), 563–576. 10.6004/jnccn.2024.0051 39413812

[B32] Siegel R. L. Kratzer T. B. Giaquinto A. N. Sung H. Jemal A. (2025). Cancer statistics, 2025. CA A cancer J clinicians. 75(1), 10–45. 10.3322/caac.21871 PMC11745215 39817679

[B33] Skrivankova V. W. Richmond R. C. Woolf B. A. R. Davies N. M. Swanson S. A. VanderWeele T. J. (2021). Strengthening the reporting of observational studies in epidemiology using mendelian randomisation (STROBE-MR): explanation and elaboration. BMJ 375, n2233. 10.1136/bmj.n2233 34702754 PMC8546498

[B34] Slob E. A. Groenen P. J. Thurik A. R. Rietveld C. A. (2017). A note on the use of egger regression in Mendelian randomization studies. Int. J. Epidemiol. 46 (6), 2094–2097. 10.1093/ije/dyx191 29025040

[B35] Verbanck M. Chen C. Y. Neale B. Do R. (2018). Detection of widespread horizontal pleiotropy in causal relationships inferred from Mendelian randomization between complex traits and diseases. Nat. Genet. 50 (5), 693–698. 10.1038/s41588-018-0099-7 29686387 PMC6083837

[B36] Wang J. Lohman G. J. S. Stubbe J. (2007). Enhanced subunit interactions with gemcitabine-5′-diphosphate inhibit ribonucleotide reductases. Proc. Natl. Acad. Sci. U. S. A. 104 (36), 14324–14329. 10.1073/pnas.0706803104 17726094 PMC1964847

[B37] Wang L. Wang W. Kaelber D. C. Xu R. Berger N. A. (2024). GLP-1 receptor agonists and colorectal cancer risk in drug-naive patients with type 2 diabetes, with and without overweight/obesity. JAMA Oncol. 10 (2), 256–258. 10.1001/jamaoncol.2023.5573 38060218 PMC10704339

[B38] Wong H. J. Sim B. Teo Y. H. Teo Y. N. Chan M. Y. Yeo L. L. L. (2025). Efficacy of GLP-1 receptor agonists on weight loss, BMI, and waist circumference for patients with obesity or overweight: a systematic review, meta-analysis, and meta-regression of 47 randomized controlled trials. Diabetes Care 48 (2), 292–300. 10.2337/dc24-1678 39841962

[B39] Zhao W. Zhang X. Zhou Z. Sun B. Gu W. Liu J. (2018). Liraglutide inhibits the proliferation and promotes the apoptosis of MCF-7 human breast cancer cells through downregulation of microRNA-27a expression. Mol. Med. Rep. 17, 5202–5212. 10.3892/mmr.2018.8475 29393459 PMC5865986

[B40] Zhao H. jun Jiang X. Hu L. juan Yang L. dong D. L. Wang Y. ping (2020). Activation of GLP-1 receptor enhances the chemosensitivity of pancreatic cancer cells. J. Mol. Endocrinol. 64 (2), 103–113. 10.1530/JME-19-0186 31855560

