# Supplementary material for: The impact of GLP-1 receptor agonists on chemotherapeutic efficacy: insights from GWAS and experiments
Source: Front Pharmacol. 2026 Oct 23;17:1957757. doi: 10.3389/fphar.2026.1957757 (PMC13741220; doi:10.3389/fphar.2026.1957757)

NC

Semaglutide

MTX

MTX + Semaglutide

PTX

PTX + Semaglutide

Gem

Gem + Semaglutide

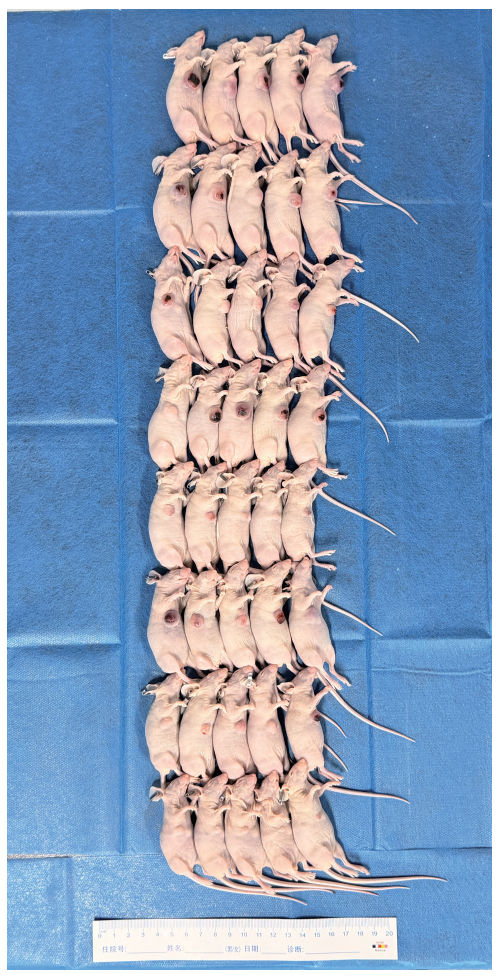

Supplement: Supplementary file 1 [file Image1.pdf]
