# Supplementary material for: The impact of GLP-1 receptor agonists on chemotherapeutic efficacy: insights from GWAS and experiments
Source: Front Pharmacol. 2026 Oct 23;17:1957757. doi: 10.3389/fphar.2026.1957757 (PMC13741220; doi:10.3389/fphar.2026.1957757)
Supplement: Supplementary file 2 [file Image10.pdf]

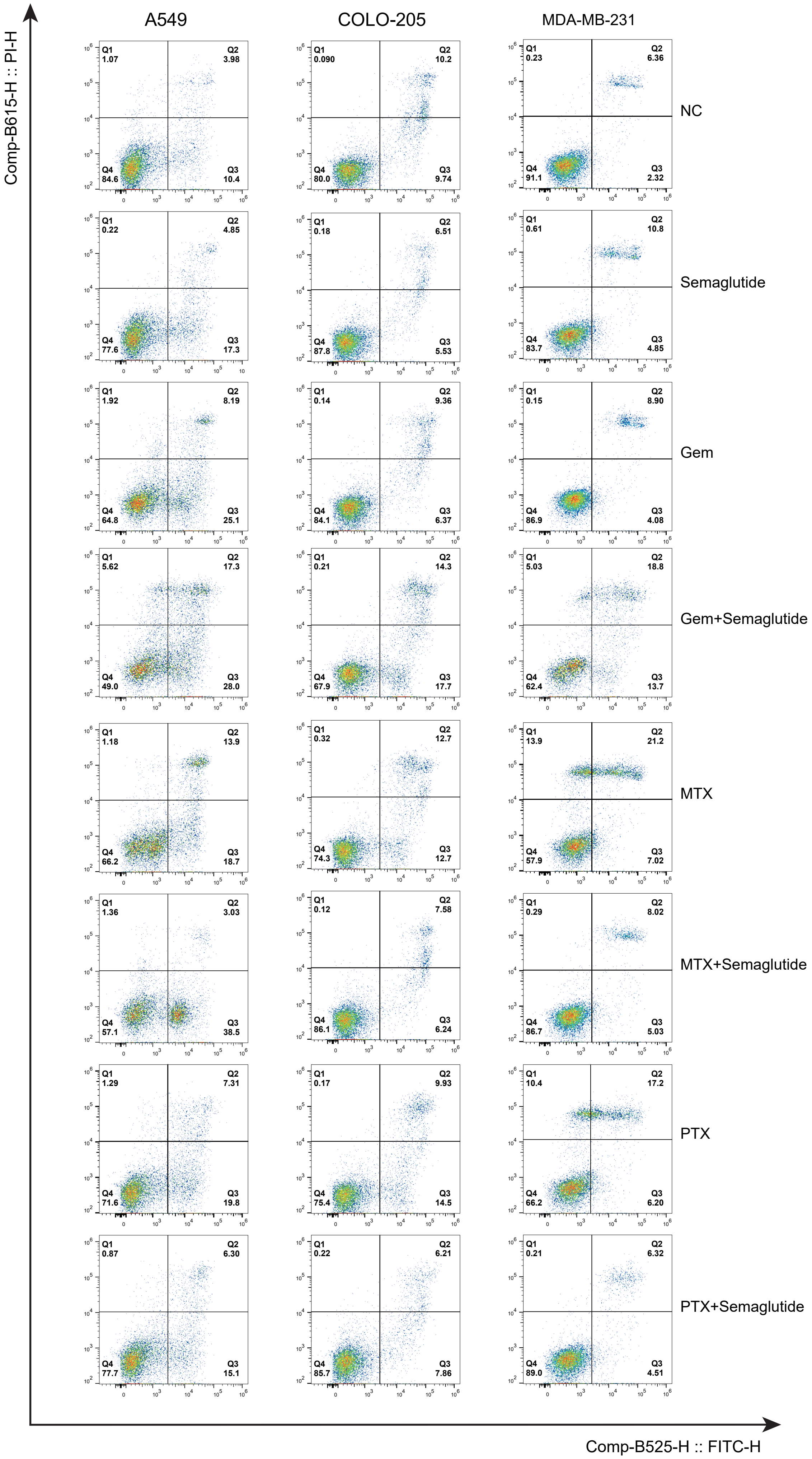

A549
COLO-205
MDA-MB-231
NC
Semaglutide
Gem
Gem+Semaglutide
MTX
MTX+Semaglutide
PTX
PTX+Semaglutide
Comp-B615-H :: PI-H
Comp-B525-H :: FITC-H
