# Supplementary material for: The impact of GLP-1 receptor agonists on chemotherapeutic efficacy: insights from GWAS and experiments
Source: Front Pharmacol. 2026 Oct 23;17:1957757. doi: 10.3389/fphar.2026.1957757 (PMC13741220; doi:10.3389/fphar.2026.1957757)
Supplement: Supplementary file 3 [file Image11.pdf]

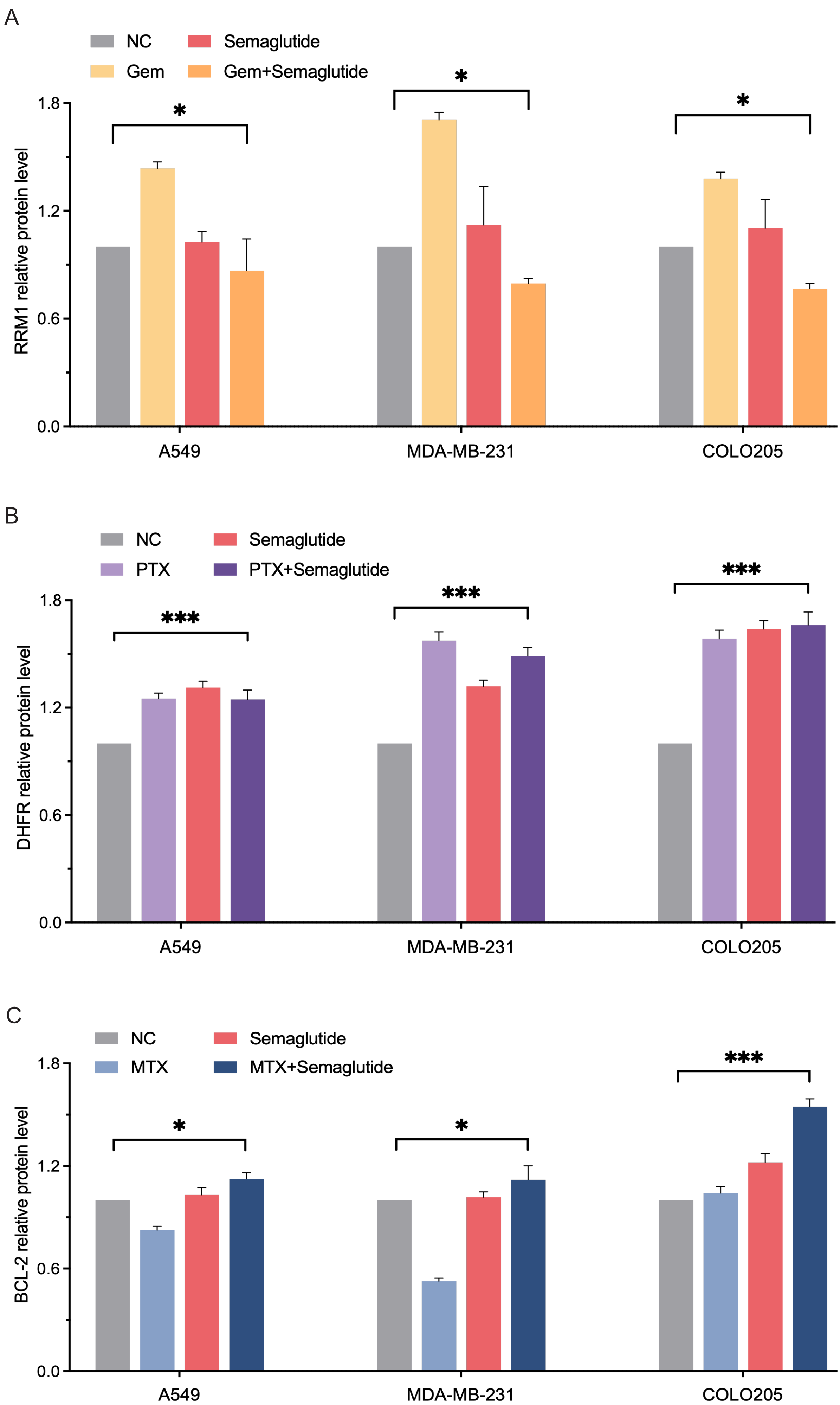
A
NC
Semaglutide
Gem
Gem+Semaglutide
RRM1 relative protein level
1.8
1.2
0.6
0.0
*
*
*
A549
MDA-MB-231
COLO205
B
NC
Semaglutide
PTX
PTX+Semaglutide
DHFR relative protein level
1.8
1.2
0.6
0.0
***
***
***
A549
MDA-MB-231
COLO205
C
NC
Semaglutide
MTX
MTX+Semaglutide
BCL-2 relative protein level
1.8
1.2
0.6
0.0
*
*
***
A549
MDA-MB-231
COLO205
