# Supplementary material for: The impact of GLP-1 receptor agonists on chemotherapeutic efficacy: insights from GWAS and experiments
Source: Front Pharmacol. 2026 Oct 23;17:1957757. doi: 10.3389/fphar.2026.1957757 (PMC13741220; doi:10.3389/fphar.2026.1957757)
Supplement: Supplementary file 4 [file Image2.pdf]

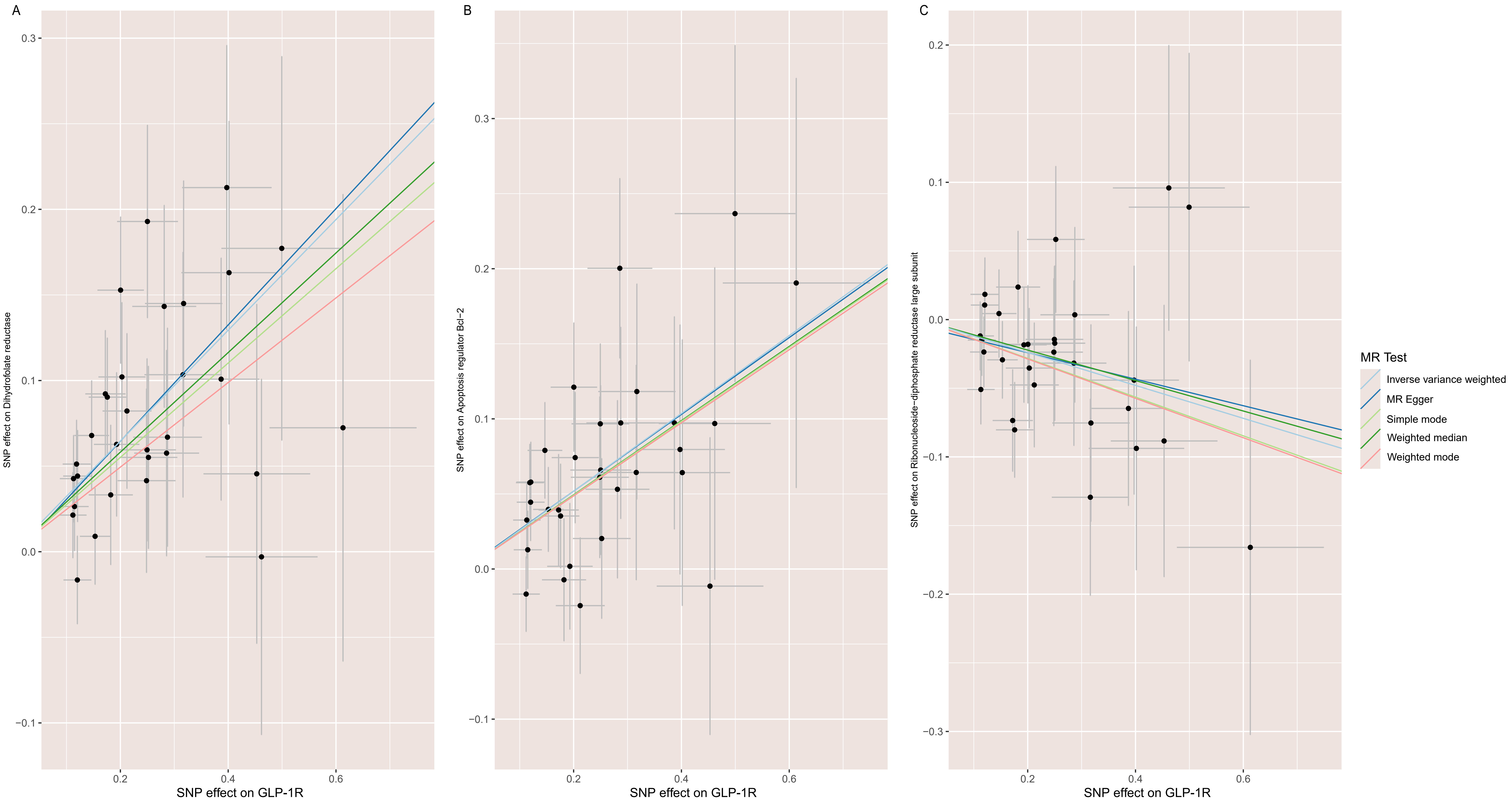

A
B
C
SNP effect on Dihydrofolate reductase
SNP effect on Apoptosis regulator Bcl−2
SNP effect on Ribonucleoside−diphosphate reductase large subunit
SNP effect on GLP-1R
0.3
0.2
0.1
0.0
−0.1
−0.2
−0.3
0.4
0.6
MR Test
Inverse variance weighted
MR Egger
Simple mode
Weighted median
Weighted mode
