# Supplementary material for: The impact of GLP-1 receptor agonists on chemotherapeutic efficacy: insights from GWAS and experiments
Source: Front Pharmacol. 2026 Oct 23;17:1957757. doi: 10.3389/fphar.2026.1957757 (PMC13741220; doi:10.3389/fphar.2026.1957757)
Supplement: Supplementary file 5 [file Image3.pdf]

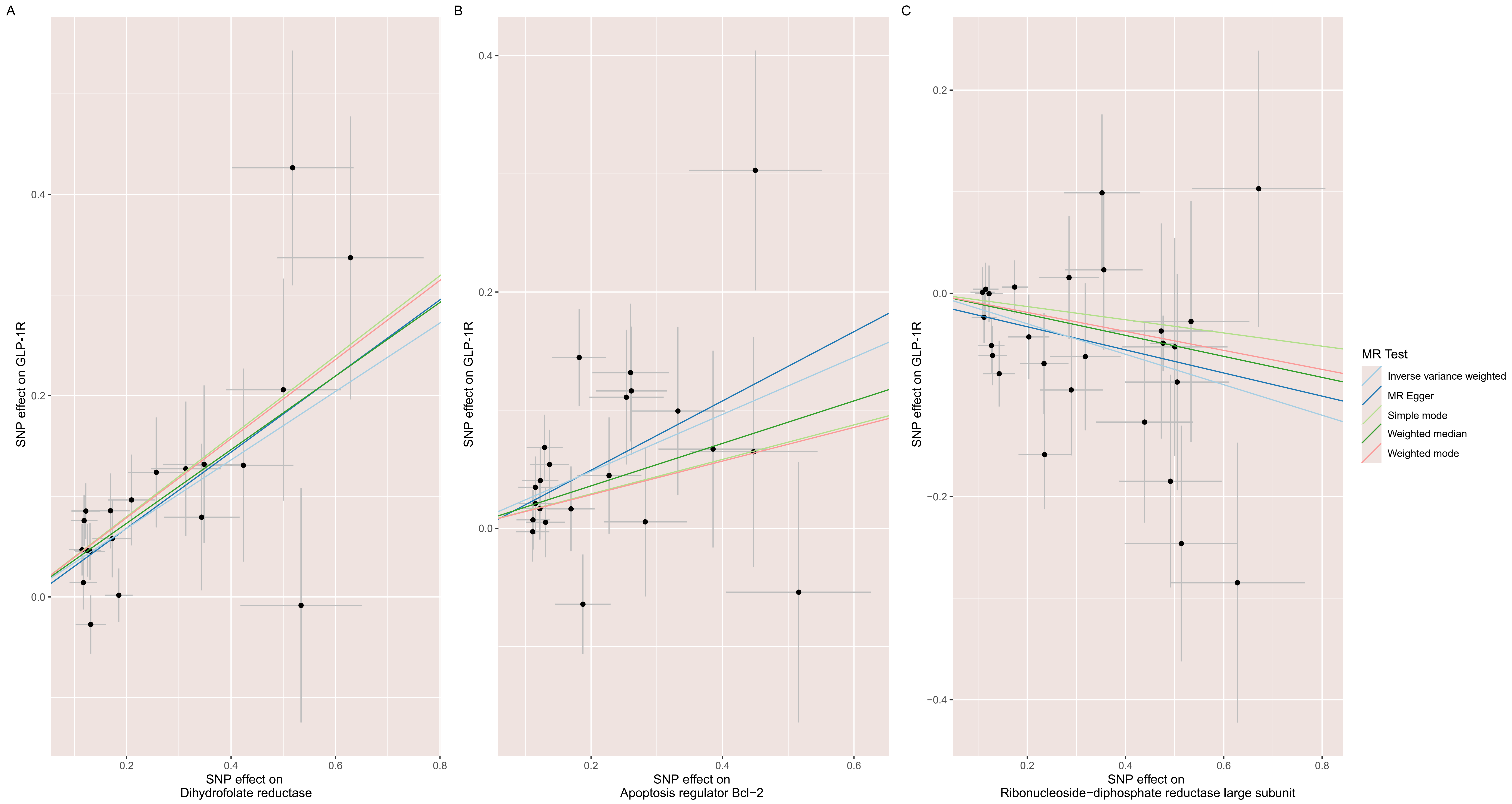
A
SNP effect on GLP-1R
SNP effect on
Dihydrofolate reductase
0.0
0.2
0.4
0.2
0.4
0.6
0.8
B
SNP effect on GLP-1R
SNP effect on
Apoptosis regulator Bcl−2
0.0
0.2
0.4
0.2
0.4
0.6
C
SNP effect on GLP-1R
SNP effect on
Ribonucleoside−diphosphate reductase large subunit
−0.4
−0.2
0.0
0.2
0.2
0.4
0.6
0.8
MR Test
Inverse variance weighted
MR Egger
Simple mode
Weighted median
Weighted mode
