# Supplementary material for: The impact of GLP-1 receptor agonists on chemotherapeutic efficacy: insights from GWAS and experiments
Source: Front Pharmacol. 2026 Oct 23;17:1957757. doi: 10.3389/fphar.2026.1957757 (PMC13741220; doi:10.3389/fphar.2026.1957757)
Supplement: Supplementary file 6 [file Image4.pdf]

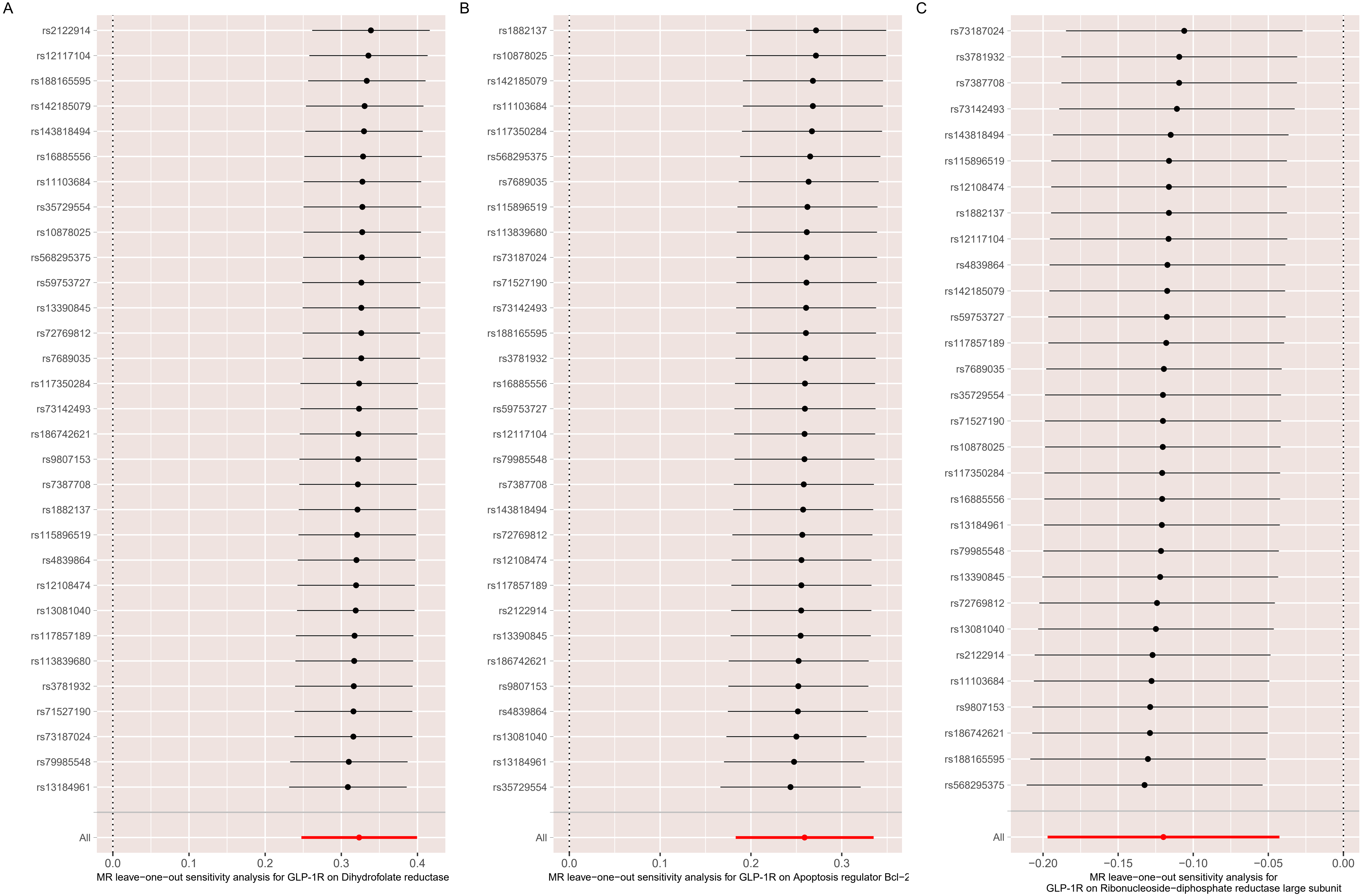

A
rs2122914
rs12117104
rs188165595
rs142185079
rs143818494
rs16885556
rs11103684
rs35729554
rs10878025
rs568295375
rs59753727
rs13390845
rs72769812
rs7689035
rs117350284
rs73142493
rs186742621
rs9807153
rs7387708
rs1882137
rs115896519
rs4839864
rs12108474
rs13081040
rs117857189
rs113839680
rs3781932
rs71527190
rs73187024
rs79985548
rs13184961
All
0.0
0.1
0.2
0.3
0.4
MR leave−one−out sensitivity analysis for GLP-1R on Dihydrofolate reductase
B
rs1882137
rs10878025
rs142185079
rs11103684
rs117350284
rs568295375
rs7689035
rs115896519
rs113839680
rs73187024
rs71527190
rs73142493
rs188165595
rs3781932
rs16885556
rs59753727
rs12117104
rs79985548
rs7387708
rs143818494
rs72769812
rs12108474
rs117857189
rs2122914
rs13390845
rs186742621
rs9807153
rs4839864
rs13081040
rs13184961
rs35729554
All
0.0
0.1
0.2
0.3
MR leave−one−out sensitivity analysis for GLP-1R on Apoptosis regulator Bcl−2
C
rs73187024
rs3781932
rs7387708
rs73142493
rs143818494
rs115896519
rs12108474
rs1882137
rs12117104
rs4839864
rs142185079
rs59753727
rs117857189
rs7689035
rs35729554
rs71527190
rs10878025
rs117350284
rs16885556
rs13184961
rs79985548
rs13390845
rs72769812
rs13081040
rs2122914
rs11103684
rs9807153
rs186742621
rs188165595
rs568295375
All
−0.20
−0.15
−0.10
−0.05
0.00
MR leave−one−out sensitivity analysis for
GLP-1R on Ribonucleoside−diphosphate reductase large subunit
