# Supplementary material for: The impact of GLP-1 receptor agonists on chemotherapeutic efficacy: insights from GWAS and experiments
Source: Front Pharmacol. 2026 Oct 23;17:1957757. doi: 10.3389/fphar.2026.1957757 (PMC13741220; doi:10.3389/fphar.2026.1957757)
Supplement: Supplementary file 7 [file Image5.pdf]

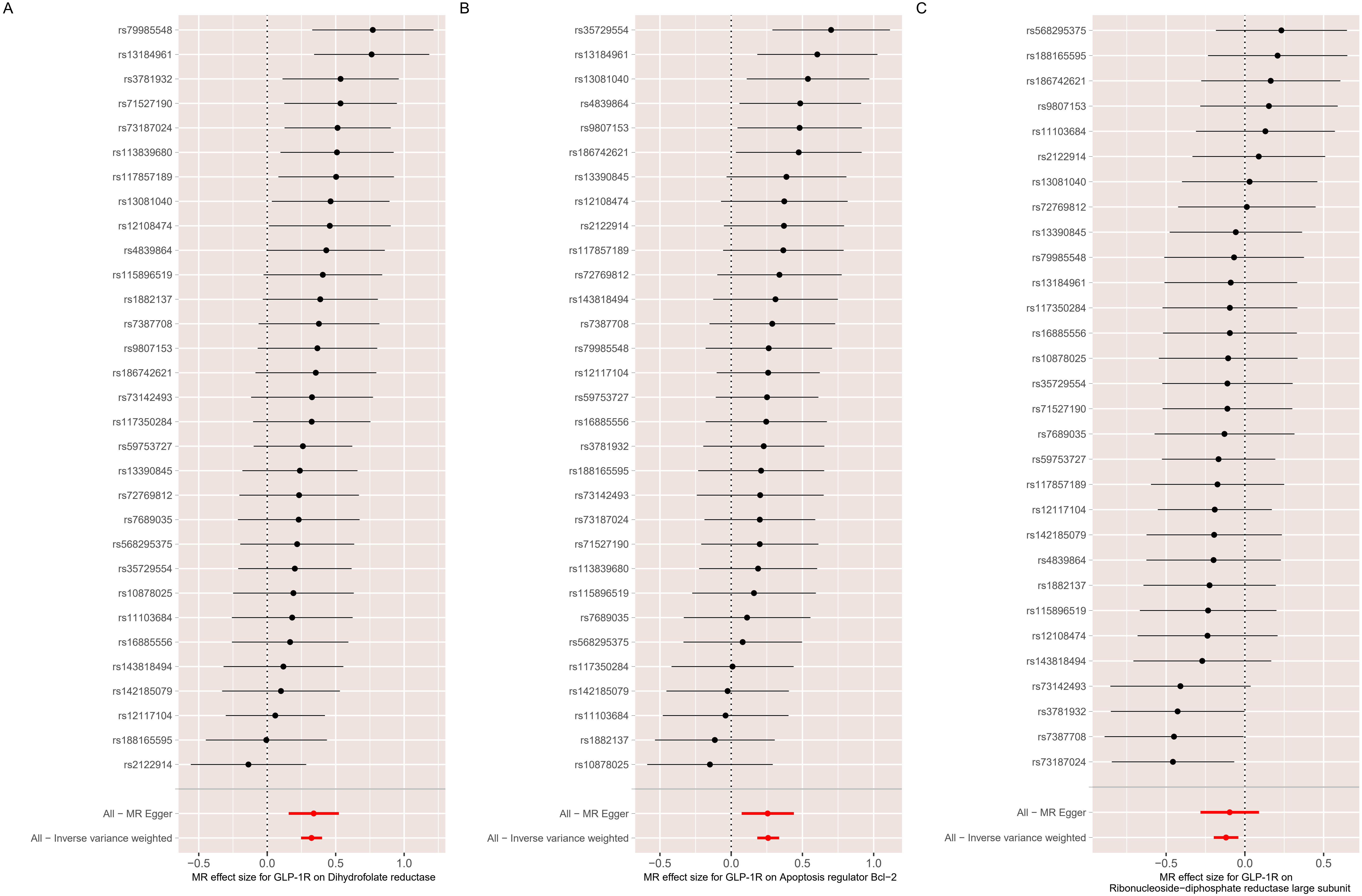

A
rs79985548
rs13184961
rs3781932
rs71527190
rs73187024
rs113839680
rs117857189
rs13081040
rs12108474
rs4839864
rs115896519
rs1882137
rs7387708
rs9807153
rs186742621
rs73142493
rs117350284
rs59753727
rs13390845
rs72769812
rs7689035
rs568295375
rs35729554
rs10878025
rs11103684
rs16885556
rs143818494
rs142185079
rs12117104
rs188165595
rs2122914
All − MR Egger
All − Inverse variance weighted
−0.5
0.0
0.5
1.0
MR effect size for GLP-1R on Dihydrofolate reductase
B
rs35729554
rs13184961
rs13081040
rs4839864
rs9807153
rs186742621
rs13390845
rs12108474
rs2122914
rs117857189
rs72769812
rs143818494
rs7387708
rs79985548
rs12117104
rs59753727
rs16885556
rs3781932
rs188165595
rs73142493
rs73187024
rs71527190
rs113839680
rs115896519
rs7689035
rs568295375
rs117350284
rs142185079
rs11103684
rs1882137
rs10878025
All − MR Egger
All − Inverse variance weighted
−0.5
0.0
0.5
1.0
MR effect size for GLP-1R on Apoptosis regulator Bcl−2
C
rs568295375
rs188165595
rs186742621
rs9807153
rs11103684
rs2122914
rs13081040
rs72769812
rs13390845
rs79985548
rs13184961
rs117350284
rs16885556
rs10878025
rs35729554
rs71527190
rs7689035
rs59753727
rs117857189
rs12117104
rs142185079
rs4839864
rs1882137
rs115896519
rs12108474
rs143818494
rs73142493
rs3781932
rs7387708
rs73187024
All − MR Egger
All − Inverse variance weighted
−0.5
0.0
0.5
MR effect size for GLP-1R on
Ribonucleoside−diphosphate reductase large subunit
