# Supplementary material for: The impact of GLP-1 receptor agonists on chemotherapeutic efficacy: insights from GWAS and experiments
Source: Front Pharmacol. 2026 Oct 23;17:1957757. doi: 10.3389/fphar.2026.1957757 (PMC13741220; doi:10.3389/fphar.2026.1957757)
Supplement: Supplementary file 8 [file Image6.pdf]

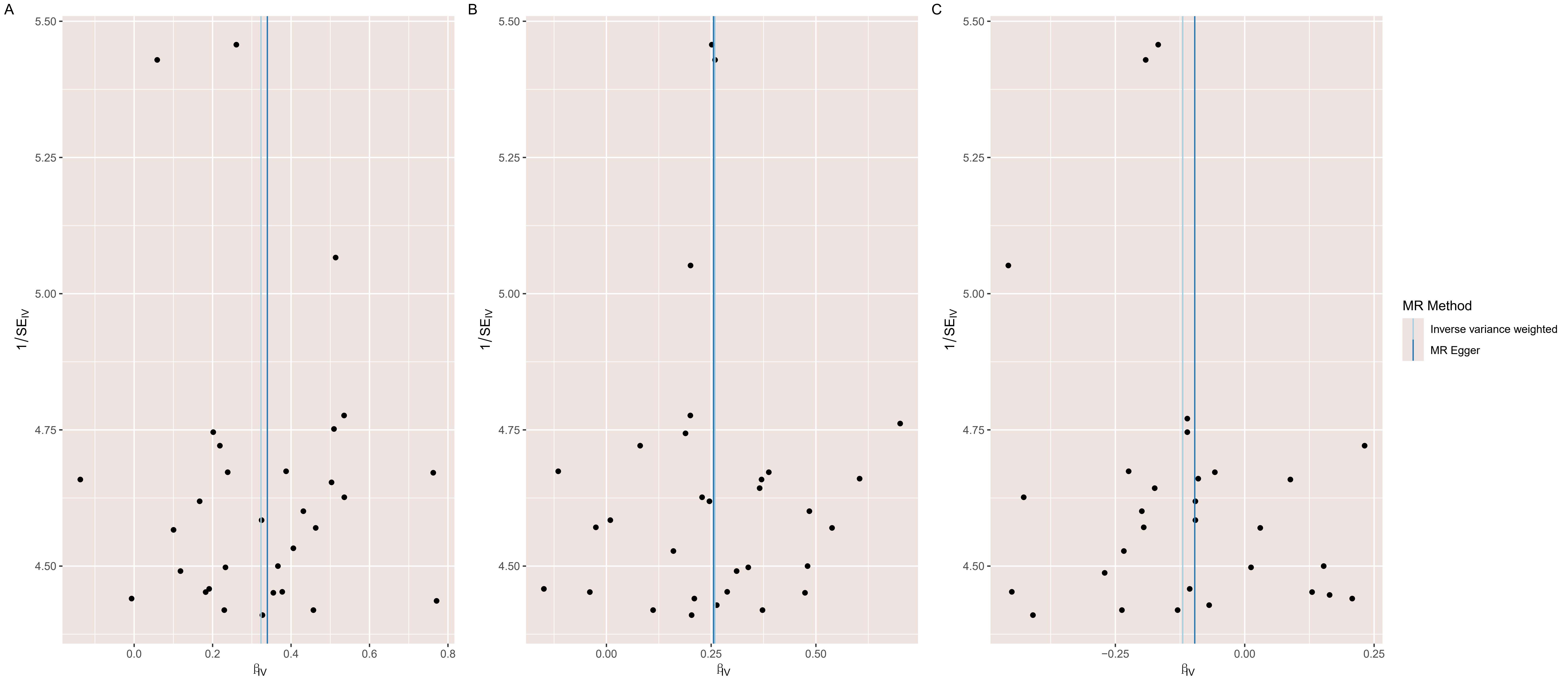
A
B
C
5.50
5.25
5.00
4.75
4.50
$1/SE_{IV}$
0.0
0.2
0.4
0.6
0.8
0.00
0.25
0.50
−0.25
β_{IV}
MR Method
Inverse variance weighted
MR Egger
