# Supplementary material for: The impact of GLP-1 receptor agonists on chemotherapeutic efficacy: insights from GWAS and experiments
Source: Front Pharmacol. 2026 Oct 23;17:1957757. doi: 10.3389/fphar.2026.1957757 (PMC13741220; doi:10.3389/fphar.2026.1957757)
Supplement: Supplementary file 9 [file Image7.pdf]

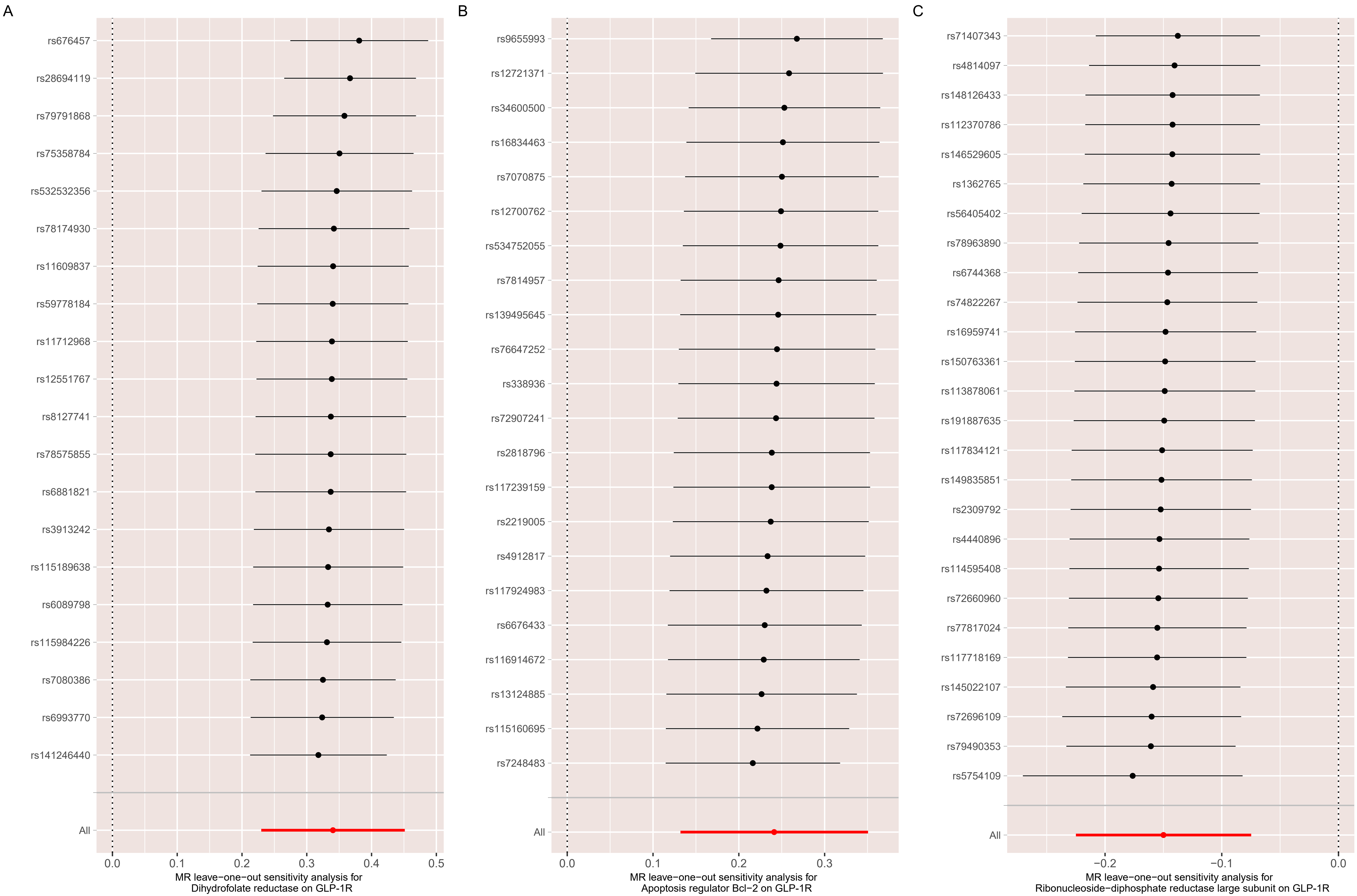
A
rs676457
rs28694119
rs79791868
rs75358784
rs532532356
rs78174930
rs11609837
rs59778184
rs11712968
rs12551767
rs8127741
rs78575855
rs6881821
rs3913242
rs115189638
rs6089798
rs115984226
rs7080386
rs6993770
rs141246440
All
0.0 0.1 0.2 0.3 0.4 0.5
MR leave−one−out sensitivity analysis for
Dihydrofolate reductase on GLP-1R
B
rs9655993
rs12721371
rs34600500
rs16834463
rs7070875
rs12700762
rs534752055
rs7814957
rs139495645
rs76647252
rs338936
rs72907241
rs2818796
rs117239159
rs2219005
rs4912817
rs117924983
rs6676433
rs116914672
rs13124885
rs115160695
rs7248483
All
0.0 0.1 0.2 0.3
MR leave−one−out sensitivity analysis for
Apoptosis regulator Bcl−2 on GLP-1R
C
rs71407343
rs4814097
rs148126433
rs112370786
rs146529605
rs1362765
rs56405402
rs78963890
rs6744368
rs74822267
rs16959741
rs150763361
rs113878061
rs191887635
rs117834121
rs149835851
rs2309792
rs4440896
rs114595408
rs72660960
rs77817024
rs117718169
rs145022107
rs72696109
rs79490353
rs5754109
All
−0.2 −0.1 0.0
MR leave−one−out sensitivity analysis for
Ribonucleoside−diphosphate reductase large subunit on GLP-1R
