# Supplementary material for: The impact of GLP-1 receptor agonists on chemotherapeutic efficacy: insights from GWAS and experiments
Source: Front Pharmacol. 2026 Oct 23;17:1957757. doi: 10.3389/fphar.2026.1957757 (PMC13741220; doi:10.3389/fphar.2026.1957757)
Supplement: Supplementary file 10 [file Image8.pdf]

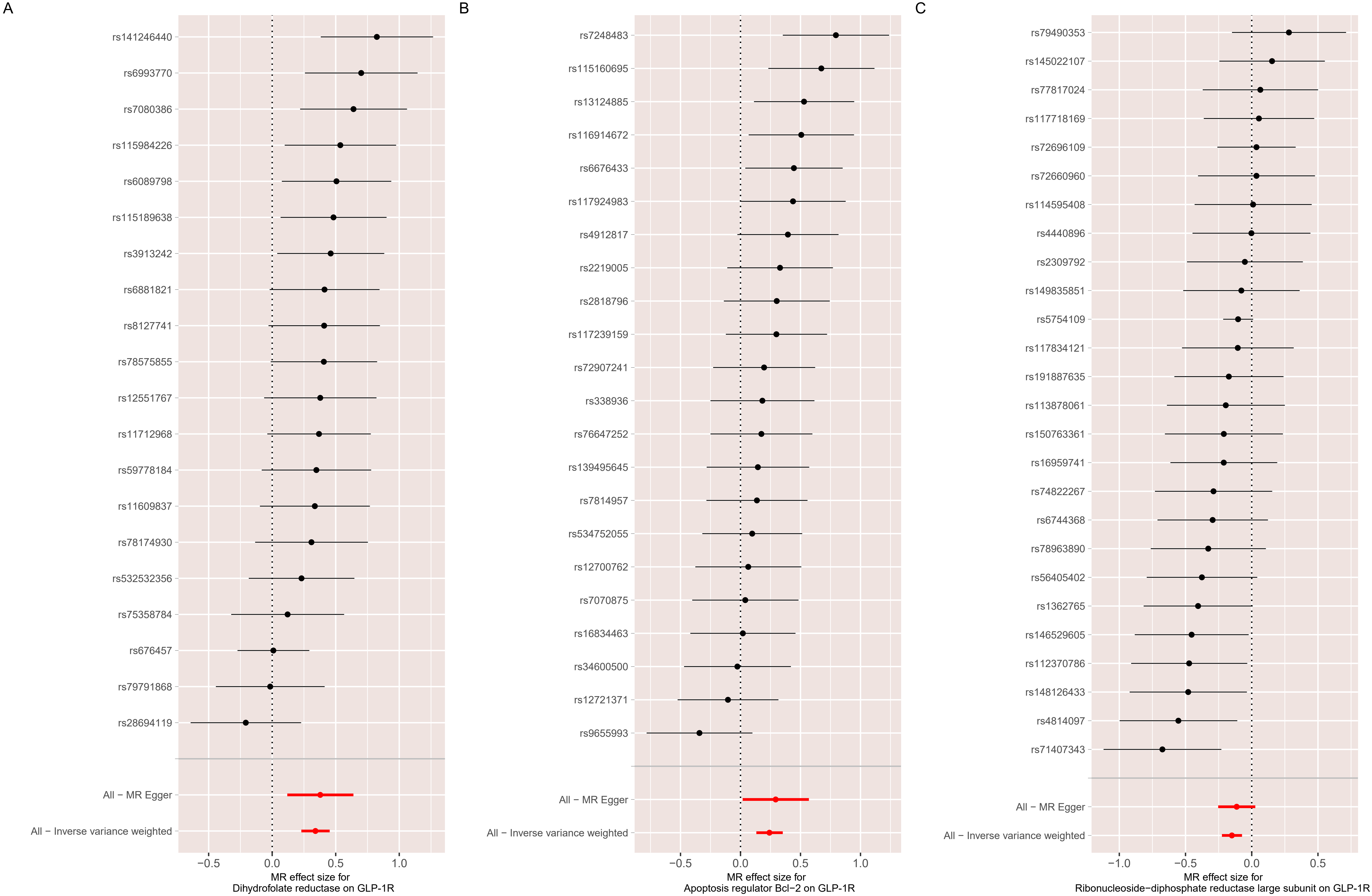
A
rs141246440
rs6993770
rs7080386
rs115984226
rs6089798
rs115189638
rs3913242
rs6881821
rs8127741
rs78575855
rs12551767
rs11712968
rs59778184
rs11609837
rs78174930
rs532532356
rs75358784
rs676457
rs79791868
rs28694119
All − MR Egger
All − Inverse variance weighted
−0.5
0.0
0.5
1.0
MR effect size for
Dihydrofolate reductase on GLP-1R
B
rs7248483
rs115160695
rs13124885
rs116914672
rs6676433
rs117924983
rs4912817
rs2219005
rs2818796
rs117239159
rs72907241
rs338936
rs76647252
rs139495645
rs7814957
rs534752055
rs12700762
rs7070875
rs16834463
rs34600500
rs12721371
rs9655993
All − MR Egger
All − Inverse variance weighted
−0.5
0.0
0.5
1.0
MR effect size for
Apoptosis regulator Bcl−2 on GLP-1R
C
rs79490353
rs145022107
rs77817024
rs117718169
rs72696109
rs72660960
rs114595408
rs4440896
rs2309792
rs149835851
rs5754109
rs117834121
rs191887635
rs113878061
rs150763361
rs16959741
rs74822267
rs6744368
rs78963890
rs56405402
rs1362765
rs146529605
rs112370786
rs148126433
rs4814097
rs71407343
All − MR Egger
All − Inverse variance weighted
−1.0
−0.5
0.0
0.5
MR effect size for
Ribonucleoside−diphosphate reductase large subunit on GLP-1R
