# Supplementary material for: The impact of GLP-1 receptor agonists on chemotherapeutic efficacy: insights from GWAS and experiments
Source: Front Pharmacol. 2026 Oct 23;17:1957757. doi: 10.3389/fphar.2026.1957757 (PMC13741220; doi:10.3389/fphar.2026.1957757)
Supplement: Supplementary file 11 [file Image9.pdf]

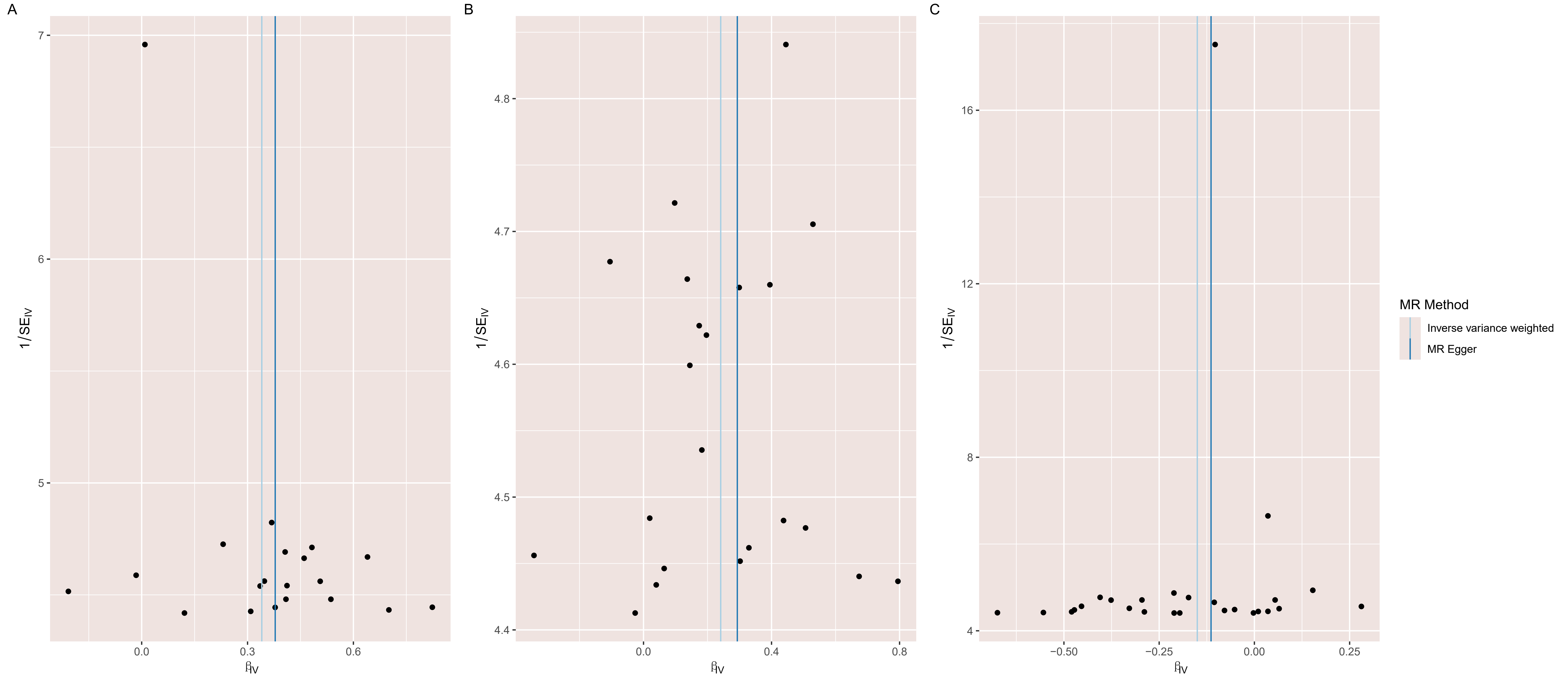
A
B
C
1/SE$_{IV}$
β_{IV}
7
6
5
0.0
0.3
0.6
4.8
4.7
4.6
4.5
4.4
0.4
0.8
16
12
8
4
−0.50
−0.25
0.00
0.25
MR Method
Inverse variance weighted
MR Egger
